# Body Roundness Index Is a Superior Obesity Index in Predicting Diabetes Risk Among Hypertensive Patients: A Prospective Cohort Study in China

**DOI:** 10.3389/fcvm.2021.736073

**Published:** 2021-11-18

**Authors:** Yingshan Liu, Xiaocong Liu, Haixia Guan, Shuting Zhang, Qibo Zhu, Xiaoying Fu, Hongmei Chen, Songtao Tang, Yingqing Feng, Jian Kuang

**Affiliations:** ^1^Department of Endocrinology, Guangdong Provincial People's Hospital, Guangdong Academy of Medical Sciences, Guangzhou, China; ^2^Department of Cardiology, Guangdong Cardiovascular Institute, Guangdong Provincial People's Hospital, Guangdong Academy of Medical Sciences, Guangzhou, China; ^3^Community Health Center of Liaobu County, Dongguan, China

**Keywords:** hypertension, diabetes, cardiovascular disease, anthropometry, central obesity, body roundness index

## Abstract

**Objective:** Individuals with both hypertension and diabetes have been confirmed to significantly increase the risk of cardiovascular disease morbidity and mortality compared with those with only hypertension or diabetes. This study aimed to evaluate the potential of different anthropometric indices for predicting diabetes risk among hypertensive patients.

**Methods:** The study group consisted of 6,990 hypertensive adults without diabetes who were recruited in China. Demographic and clinical assessment, physical examinations, laboratory tests, and anthropometric measurements, including body mass index (BMI), waist circumference (WC), hip circumference (HC), waist-to-hip ratio (WHR), waist-to-height ratio (WHtR), and novel indices (ABSI, AVI, BAI, BRI, CI, WWI, and WHHR), were performed at baseline and during the (median) 3-year follow-up. Cox regression analyses were conducted to estimate effects from these indices for the onset of diabetes. Receiver operator characteristic (ROC) analyses were conducted to assess the predictive capacities of the anthropometric indices and determine the optimal cut-points.

**Results:** A total of 816 (11.7%) developed diabetes during our prospective study. Multivariate Cox regression analyses revealed weight, WC, WHR, WHtR, BAI, BRI, and WWI as the independent risk factor for diabetes among hypertensive patients, regardless of whether it was treated as a continuous or categorical variable (*P* < 0.05). Further Cox analyses combining BMI and different central obesity indices showed that elevated WC, WHR, WHtR, AVI, BRI, CI, regardless of the general obesity status, were found to be each independently associated with increased diabetes risk (*P* < 0.05). Dynamic increases of BRI < 5.24 to BRI ≥ 5.24 were associated with increased risk (HR = 1.29; 95% CI, 1.02, 1.64), and its reversal was associated with reduced risk (HR = 1.56; 95% CI, 1.23, 1.98) compared with the others (HR = 1.95; 95% CI, 1.63, 2.32). ROC analysis indicated that the areas under the ROC curves (AUC) of the anthropometric indices ranged from 0.531 to 0.63, with BRI (cut-off value = 4.62) and WHtR having the largest area.

**Conclusions:** Based on this novel study, BRI was the most superior predictor and independent determinant for diabetes onset among the hypertensive population. Hypertensive patients with BRI > 4.62, regardless of general obesity status, were at high risk of diabetes. Thus, the prompt screening and diagnosis of diabetes should be carried out among these patients for timely integrated intervention.

## Introduction

The global burden of diabetes and hypertension is tremendous and increasing continuously (1). Globally, around 422 million and 1.13 billion people are suffering from diabetes and hypertension, respectively. Both diabetes and hypertension are substantial risk factors for cardiovascular disease (CVD) morbidity and mortality. Diabetes and hypertension frequently coexist (2), suffering from both diseases significantly evaluate the morbidity and mortality of CVD compared with those with either condition alone (3). A 2-fold increase in CVD risk has been seen in individuals with both diabetes and hypertension compared with the hypertensive patients without diabetes. Hypertension could be easily identified by non-invasive BP measurements, yet diabetes often goes undetected until patients present with diabetic complications. Therefore, early recognition of hypertensive patients at high risk of diabetes may result in improved prevention and early detection.

Hypertension is characterized by increased peripheral vascular resistance and endothelial dysfunction, and diabetes is characterized by insulin resistance and β-cell dysfunction (4). These pathophysiological processes intercommunicate tightly in various ways, of which obesity act as an important confounder of the association between blood pressure and blood sugar since it is an established risk factor for both diabetes and hypertension (1, 5). More importantly, obesity is a reversible predisposing factor for these two conditions. There is considerable evidence to show that weight loss can reduce or delay the onset of diabetes among the high-risk population (6). Obesity mainly represents two main subtypes, general obesity, and central obesity. Recent studies revealed that BMI poorly performed in predicting diabetes, CVD, and death (7, 8). This may be explained by the characteristics of body composition in diabetes, including the increase in total fat mass (9, 10), and decrease of muscle mass or bone density, which could lead to a normal BMI even with an increase in fat mass. Moreover, mounting evidence has confirmed that central obesity is more closely correlated with insulin resistance, diabetes, and CVD than general obesity (11). Waist circumference (WC) is commonly used to define central obesity, which shows a good correlation with abdominal fat and CVD risk (12). However, WC is easily affected by the differences in height. Consequently, waist-to-hip ratio (WHR) and waist-to-height ratio (WHtR) have been developed and studied as alternatives to WC (13). Several recent studies have shown the superiority of WHtR and WHR, especially WHtR, over WC in predicting cardiometabolic diseases, while others have shown no obvious difference between them (14, 15). Additionally, some novel anthropometric indices, such as a body shape index (ABSI) (16), abdominal volume index (AVI) (17), body adiposity index (BAI) (18), body roundness index (BRI) (19), conicity index (CI) (20), weight-adjusted-waist-index (WWI) (21), and waist-hip-height ratio (WHHR) (22), have been applied as measures of adiposity. Anthropometry is a widely used, inexpensive, simple, and easy technique. Digging out the anthropometric index that is most strongly related to the occurrence of diabetes in hypertensive patients has significant clinical and public health significance. However, the relationships between different anthropometric indices with the occurrence of diabetes in Asian hypertensive patients are still scarce, and most of the available published clinical literature are cross-sectional designed and exhibit a lack of concern for the population of non-general obesity but with central obesity.

This study aimed to examine in detail the anthropometric indices in the assessment of diabetes among the Chinese hypertensive population. We compared the association of baseline and changing trends of different anthropometric indices, as well as the combinations of BMI and the indices of central obesity with diabetes risk. The predictive performances of these indices for pre-screening of diabetes were also examined.

## Methods

### Study Design and Study Population

**Figure 1:**
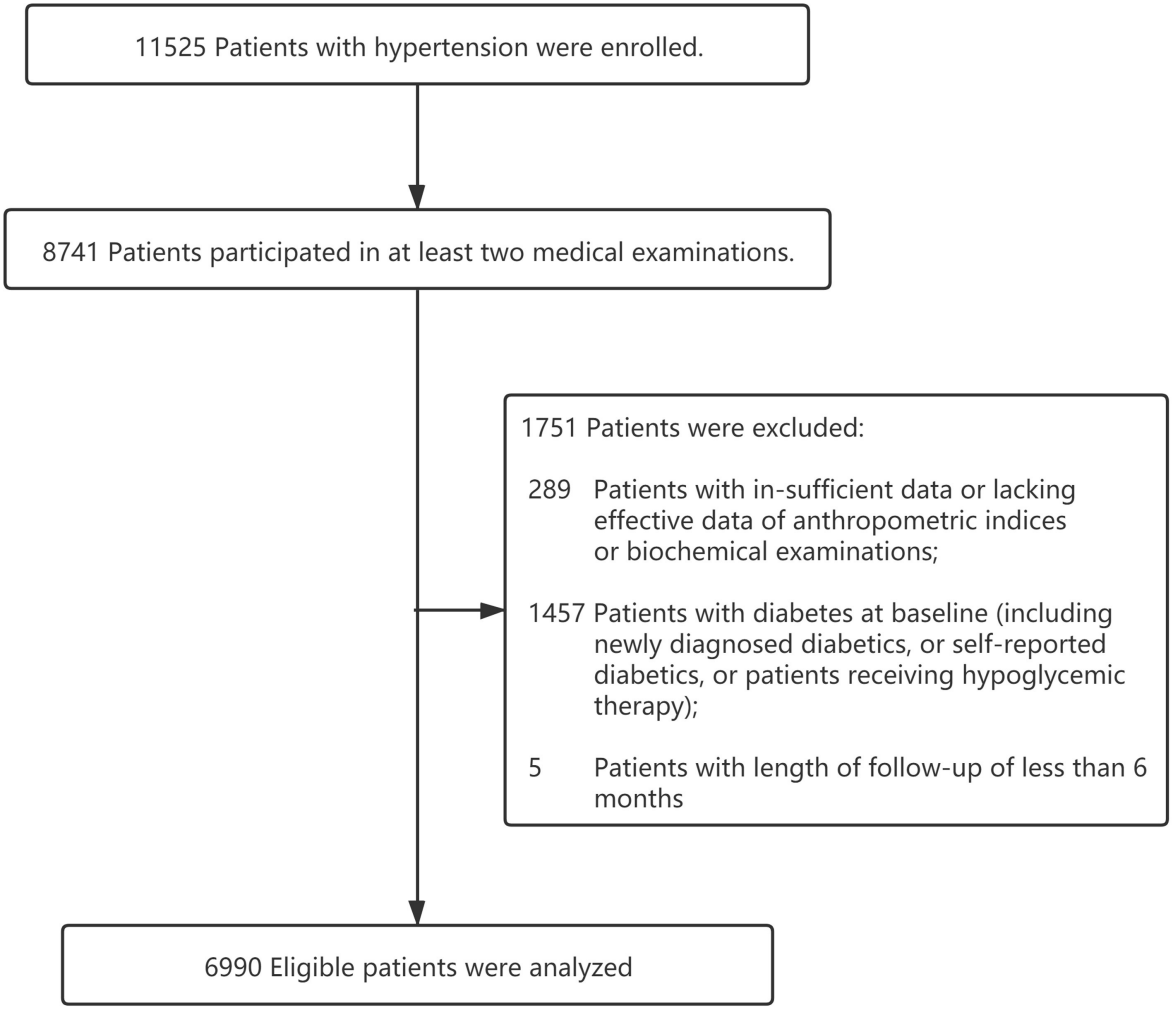
Flow chart of the study population.

The present study was based on a prospective cohort design. All participants were recruited from Dongguan City, a medium-developed and representative urbanized area of China, from 2012 to 2015. Participants needed to meet the following inclusion criteria: (1) patients with a definitively diagnosed hypertension; (2) a minimum of 18 years of age; (3) willingness to do at least 1-year follow-up; (4) not currently pregnant; (5) without cancer or other serious diseases. Exclusion criteria were as follows: (1) lacking effective data of anthropometric indices or biochemical examinations; (2) length of follow-up of <6 months; (3) history of diabetes prior to the study start date (Figure 1). Finally, a total of 6,990 hypertensive patients were included. This study was performed in accordance with the Declaration of Helsinki and was approved by the Medical Research Ethics Committee of Guangdong Provincial People's Hospital. All participants provided written informed consent before voluntary participation.

### Health Screening Questionnaire

All participants were required to complete a structured modified health screening questionnaire to determine their demographic characteristics, including age, sex, ethnicities, the current medication use of hypertension, and lifestyles, including smoking status and drinking status.

### Health Screening Measurements

Professional medical staff measured anthropometric indices, with participants wearing thin clothing with no footwear. Bodyweight, height, WC, and HC were measured according to standard protocols (23). Using these parameters, we evaluated other anthropometric indices, including BMI, WHR, WHtR, ABSI, AVI, BAI, BRI, CI, WWI, WHHR, according to the published formula (Supplementary Table 1).

Overweight and obesity were defined as BMI ≥ 24 kg/m^2^ and ≥ 28 kg/m^2^ according to BMI criteria established by the Working Group on Obesity in China (WGOC) (24), while abdominal obesity was defined as WC ≥ 90 cm in men or ≥ 85 cm in women according to Standards of care for type 2 diabetes in China, or WHR ≥0.90 in men or ≥0.85 in women according to WHO guidelines (25). The elevated WHtR was defined as ≥0.5 (26). Lacking uniform classification criteria, novel anthropometric indices (ABSI, AVI, BAI, BRI, CI, WWI, and WHHR) were divided into quartiles, and cut-points for these indices were initially selected at the level of 75% according to the distribution characteristics of BMI in the studied populations (Supplementary Table 2).

Blood pressure was measured after quiet sitting for 5 min. Hypertension was defined as systolic blood pressure (SBP) ≥ 140 mmHg or diastolic blood pressure (DBP) ≥ 90 mmHg, or with a self-reported history of hypertension, or use of antihypertensive medications (27).

The health screening measurements mentioned above would be measured at baseline and each annual follow-up.

### Evaluation of Laboratory Parameters

Blood and urine samples were collected in the morning after an overnight fast for at least 8 h. Fasting plasma glucose (FPG), Serum triglycerides (TG), total cholesterol (TC), high-density lipoprotein (HDL), low-density lipoprotein (LDL), uric acid (UA), creatinine (Cr), and urinary albumin excretion rate (UAER) were measured *via* a biochemical autonomic analyzer (OLYMPUS, Tokyo, Japan). The estimated glomerular filtration rate (eGFR) was calculated using the CKD-EPI creatinine equation (28).

The laboratory parameters mentioned above would be measured at baseline and each annual follow-up.

### Clinical Outcome

Incident diabetes was the endpoint of the present study. Diabetes was diagnosed adopting World Health Organization (WHO) 1999 diagnosis criteria of diabetes (29), mainly defined as an elevated fasting plasma glucose (>7.0 mmol/L), self-reported previous diagnosis of diabetes by the physician, and/or current use of hypoglycemic medication. All patients were followed until the earliest date of the following: the incident diabetes or the last follow-up date.

### Statistical Analyses

As estimated in PASS software version 15 (IBM Corp, Armonk, NY, USA), 1,168 samples would be needed in a Cox regression of the log hazard ratio (HR) to provide 90% power at a.05 significance level to detect a regression coefficient equal to 0.20 under an overall event rate of 0.10. Data are presented as *M* ± *SD* (normal distribution) or median (first quartile and third quartile) (non-normal distribution) for continuous variables, and as frequency (percentages) for categorical variables. Differences among the groups were evaluated by the Student's *t*-test (normal distribution) and by the Kruskal-Wallis rank-sum test (non-normal distribution) for continuous variables, and the chi-square tests for categorical variables. Univariate Cox regression models were applied to evaluate the association of demographic, biochemical, and clinical characteristics, and anthropometric indices with diabetes. The independent effect of baseline and dynamic changes of each anthropometric index on the risk of diabetes was estimated using multivariate Cox regression models. Two models with different sets of covariates were fitted. Stratified and interaction analyses were also conducted to evaluate the potential interactions between BRI and demographic, biochemical and clinical characteristics, and other anthropometric indices. The area under receiver operating characteristic (ROC) curves was calculated to evaluate the abilities of the anthropometric indices to predict diabetes to determine the optimal cut-off point of these indices. Subgroup ROC analyses were further performed for different gender and the menopausal status of women (49 years old was chosen as a cut-off to divide women into pre-menopausal and post-menopausal). All of the statistical analyses were conducted using the statistical software packages R version 4.0.3 (R Foundation for Statistical Computing, Vienna, Austria).

## Results

### Baseline Characteristics of the Participants

Baseline characteristics of the participants were presented in Table 1. An overall 6,990 subjects (3,467 [49.6%] men, the average age of 59.0 ± 13.9 years) were studied. During the average follow-up of 3.1 years, a total of 816 hypertensive patients developed diabetes (the baseline characteristic stratified by sex are presented in Supplementary Table 3). The levels of the anthropometric indices, including weight, BMI, WC, HC, WHR, WHtR, ABSI, AVI, BAI, BRI, CI, WWI, and WHHR, were significantly higher in subjects with diabetes (*P* < 0.05). Besides, compared with subjects without diabetes, patients with diabetes were older, had a higher proportion of women, had higher values of FPG, TG, TC, UAER, and SBP, and with a lower eGFR and rate of drinking (*P* < 0.05).

**Table 1:** Baseline characteristics between subjects with and without diabetes.

	**Total (*n* = 6,990)**	**New-onset diabetes (*n* = 816)**	**Non-diabetes (*n* = 6,174)**	***P*-value**
Age (years)	59.0 ± 13.9	61.1 ± 13.6	58.7 ± 14.0	<0.001^***^
Sex (male, n [%])	3,467 (49.6)	357 (43.8)	3,110 (50.4)	<0.001^***^
Smoking (n [%])	1,698 (24.3)	188 (23.1)	1,510 (24.5)	0.385
Drinking (n [%])	885 (12.7)	83 (10.2)	802 (13.0)	0.024^*^
SBP (mmHg)	139.25 ± 17.05	141.38 ± 17.56	138.97 ± 16.97	<0.001^***^
DBP (mmHg)	85.30 ± 11.10	85.12 ± 11.65	85.32 ± 11.03	0.641
**Laboratory examination**				
FPG (mmol/L)	4.90 ± 0.64	5.39 ± 0.73	4.83 ± 0.59	<0.001^***^
TG (mmol/L)	1.53 (1.09–2.21)	1.84 (1.32–2.75)	1.49 (1.06–2.16)	<0.001^***^
TC (mmol/L)	5.14 ± 1.12	5.18 ± 1.17	5.14 ± 1.11	0.369
HDL (mmol/L)	1.31 ± 0.35	1.30 ± 0.41	1.31 ± 0.35	0.290
LDL (mmol/L)	2.85 ± 0.81	2.88 ± 0.85	2.84 ± 0.80	0.197
eGFR (ml/[min·1.73 m^2^])	85.95 ± 21.84	84.06 ± 21.43	86.20 ± 21.88	0.009^**^
UAER (mg/24 h)	23.50 (10.90–71.40)	34.80 (16.25–126.70)	22.75 (10.60–67.31)	<0.001^***^
**Anthropometric indices**				
Weight (kg)	63.20 ± 11.94	65.85 ± 12.55	62.86 ± 11.82	<0.001^***^
BMI (kg/m^2^)	25.10 ± 3.65	26.47 ± 4.04	24.92 ± 3.55	<0.001^***^
WC (cm)	87.34 ± 9.19	90.72 ± 9.5	86.90 ± 9.04	<0.001^***^
HC (cm)	95.17 ± 7.69	97.29 ± 8.58	94.89 ± 7.52	<0.001^***^
WHtR	0.55 ± 0.06	0.58 ± 0.06	0.55 ± 0.06	<0.001^***^
WHR	0.92 ± 0.06	0.93 ± 0.08	0.92 ± 0.06	<0.001^***^
ABSI	0.081 ± 0.006	0.082 ± 0.006	0.081 ± 0.006	0.012^*^
AVI	15.49 ± 3.20	16.70 ± 3.45	15.33 ± 3.13	<0.001^***^
BAI	29.97 ± 4.92	31.47 ± 5.50	29.77 ± 4.81	<0.001^***^
BRI	4.47 ± 1.30	5.01 ± 1.43	4.40 ± 1.26	<0.001^***^
CI	1.27 ± 0.09	1.29 ± 0.09	1.27 ± 0.09	<0.001^***^
WWI	11.06 ± 0.89	11.25 ± 0.88	11.03 ± 0.89	<0.001^***^
WHHR	0.58 ± 0.05	0.59 ± 0.06	0.58 ± 0.05	<0.001^***^
**Medication**				
Hypotensive drugs (n [%])	4,726 (67.6)	4,175 (67.6)	551 (67.5)	0.955
ACEI/ARB (n [%])	3,518 (50.3)	3,113 (50.4)	405 (49.6)	0.672
Beta-receptor blocker (n [%])	637 (9.1)	551 (8.9)	86 (10.5)	0.132
CCB (n [%])	2,626 (37.6)	2,310 (37.4)	316 (38.7)	0.468
Diuretic (n [%])	562 (8.0)	502 (8.1)	60 (7.4)	0.442
Others (n [%])	107 (1.5)	90 (1.5)	17 (2.1)	0.171

*Continuous data are shown as the mean ± SD or median (Q1–Q3), and categorical data as n (%)*.

*FPG, fasting plasma glucose; TG, triglycerides; TC, total cholesterol; HDL, high-density lipoprotein; LDL, low-density lipoprotein; UA, uric acid; Scr, serum creatinine; eGFR, estimated glomerular filtration rate; BMI, body mass index; WC, waist circumference; HC, hip circumference; WHtR, waist-to-height ratio; WHR, waist-to-hip ratio; ABSI, A body shape index; AVI, abdominal volume index; BAI, body adiposity index; BRI, body roundness index; CI, conicity index; WWI, weight-adjusted-waist index; WHHR, waist-hip-height ratio; SBP, systolic blood pressure; DBP, diastolic blood pressure; ACEI, angiotensin-converting enzyme inhibitors; ARB, Angiotensin Receptor Blocker; CCB, Calcium Channel Blockers*.

* *P < 0.05*;

** *P < 0.01*;

*** *P < 0.001*.

### Correlations Between Various Baseline Anthropometric Indices and Diabetes Among Hypertensive Patients

Correlations between various baseline clinical variables and diabetes are displayed in Supplementary Table 4. Univariate Cox regression analysis revealed that diabetes was positively correlated with age, TG, TC, LDL, UA, UAER, weight, BMI, WC, WHR, WHtR, WHR, ABSI, AVI, BAI, BRI, CI, WWI, and WHHR among the hypertensive patients (*P* < 0.05).

**Figure 2:**
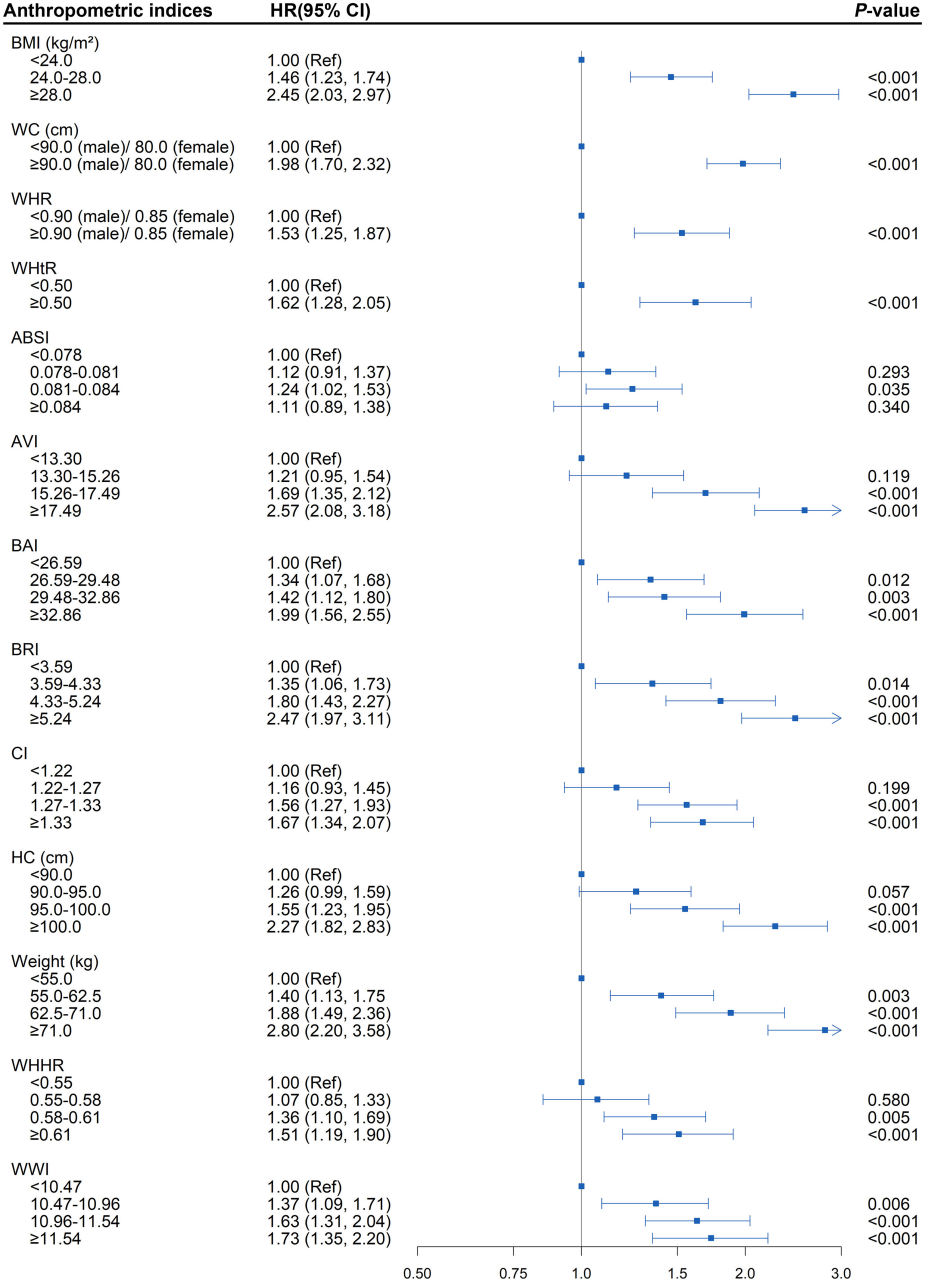
Association between separate anthropometric indices with diabetes (body mass index [BMI], waist circumference [WC], waist-to-hip ratio [WHR], waist-to-height ratio [WHtR], a body shape index [ABSI], abdominal volume index [AVI], body adiposity index [BAI], body roundness index [BRI], conicity index [CI], hip circumference [HC], weight, waist-hip-height ratio [WHHR], weight-adjusted-waist index [WWI]). The correlation was assessed by multivariate cox regression analysis, adjusted by sex, age, smoking status, drinking status, fasting plasma glucose, serum triglycerides, total cholesterol, high-density lipoprotein, low-density lipoprotein, systolic blood pressure, and diastolic blood pressure at baseline. Hazard ratios (HRs) of the anthropometric indices were represented as the squares and 95% confidence intervals (CIs) by the lines through the squares.

As shown in Supplementary Table 5, after fully adjusted for baseline age, sex, smoking status, drinking status, serum lipid levels, and blood pressure, multivariate Cox regression analysis revealed various anthropometric indices, weight (HR = 1.50; 95% CI, 1.38, 1.62), BMI (HR = 1.40; 95% CI, 1.31, 1.49), WC (HR = 1.42; 95% CI, 1.33, 1.52), HC (HR = 1.30; 95% CI, 1.22, 1.38), WHtR (HR = 1.39; 95% CI, 1.30, 1.49), WHR (HR = 1.16; 95% CI, 1.12, 1.21), AVI (HR = 1.40; 95% CI, 1.31, 1.49), BAI (HR = 1.27; 95% CI, 1.18, 1.37), BRI (HR = 1.36; 95% CI, 1.28, 1.45), CI (HR = 1.18; 95% CI, 1.10, 1.27), WWI (HR = 1.17; 95% CI, 1.08, 1.27), and WHHR (HR = 1.17; 95% CI, 1.10, 1.24), as the independent risk factor for diabetes among hypertensive patients (all *P* < 0.001). In addition, the risk of incident diabetes was found to be increased steadily with successively elevated levels of weight, BMI, WC, WHR, WHtR, BAI, BRI, and WWI (all *P* < 0.05; Figure 2). Namely, in the fully adjusted model, levels of weight, BMI, WC, WHR, WHtR, BAI, BRI, and WWI, were each associated with increased risk of diabetes, regardless of whether it was treated as a continuous or categorical variable.

**Figure 3:**
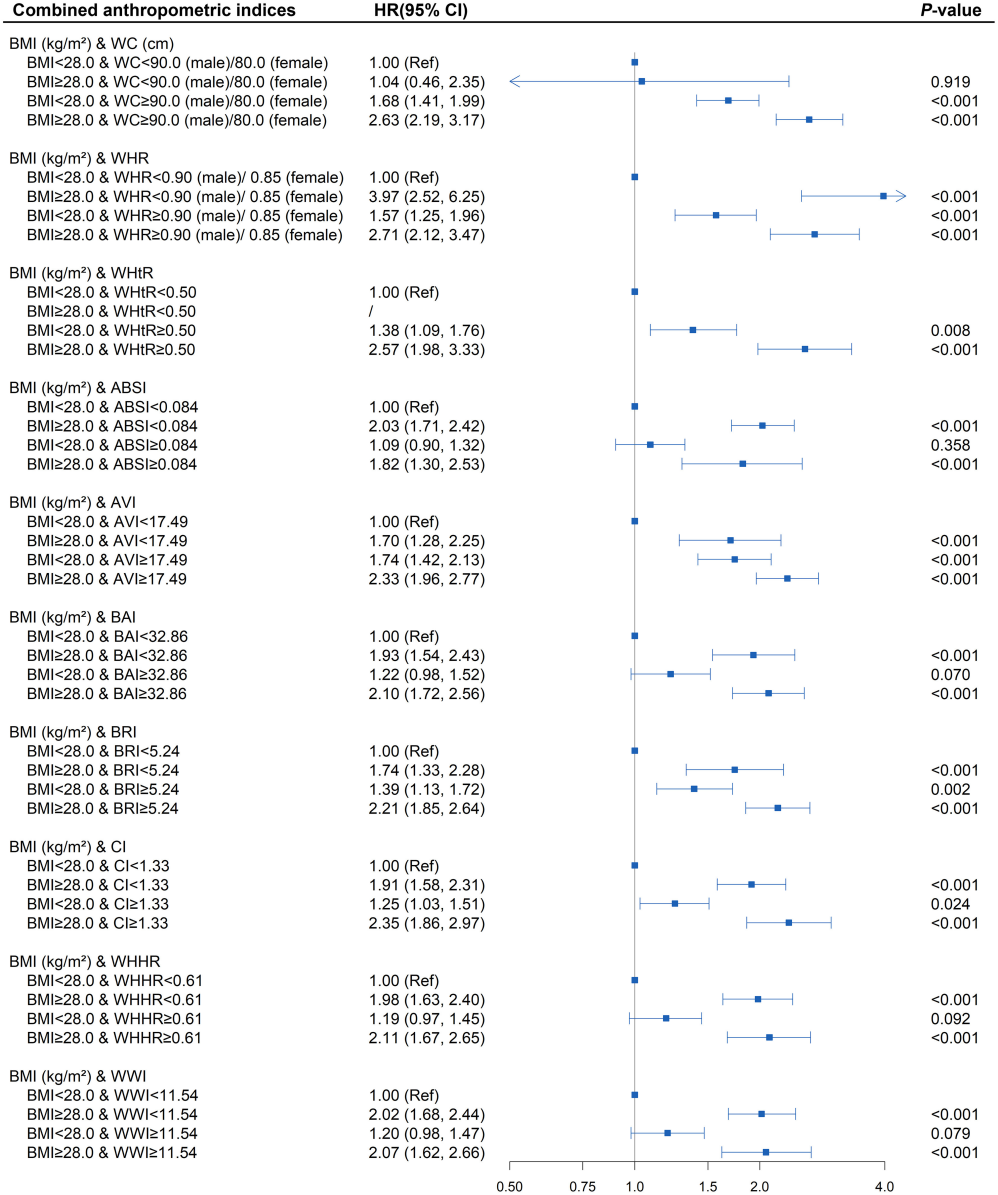
Association between different combinations of body mass index (BMI) and anthropometric indices of central obesity (waist circumference [WC], waist-to-hip ratio [WHR], waist-to-height ratio [WHtR], a body shape index [ABSI], abdominal volume index [AVI], body adiposity index [BAI], body roundness index [BRI], conicity index [CI], waist-hip-height ratio [WHHR], weight-adjusted-waist index [WWI]) with diabetes. The correlation was assessed by multivariate cox regression analysis, adjusted by sex, age, smoking status, drinking status, fasting plasma glucose, serum triglycerides, total cholesterol, high-density lipoprotein, low-density lipoprotein, systolic blood pressure, and diastolic blood pressure at baseline. Hazard ratios (HRs) of the combined anthropometric indices were represented as the squares and 95% confidence intervals (CIs) by the lines through the squares.

To better examine the performance of the central obesity indices in predicting diabetes risk, we further assessed whether the combination of BMI and different central obesity indices could better stratify the hypertensive patients with a high risk of diabetes (Supplementary Table 6 and Figure 3). The presence of elevated WC, WHR, WHtR, AVI, BRI, and CI at baseline, regardless of the general obesity status, were found to be each independently associated with increased diabetes onset risk in hypertensive patients (all *P* < 0.05). The HR (95% CI) of elevated BRI without general obesity group and elevated BRI with general obesity group were 1.74 (1.33, 2.28), 1.39 (1.13, 1.72), and 2.21 (1.85, 2.64), respectively (all *P* < 0.01). Additionally, similar to BRI, the highest risk of diabetes was all observed among the hypertensive patients with the elevated anthropometric indices mentioned above in obese states (*P* < 0.001).

Interaction and stratified analyses revealed no significant interaction between BRI and age, sex, serum lipid levels, blood pressure, smoking, and drinking status (Supplementary Table 7).

### Correlations Between Dynamic Changes of Various Anthropometric Indices and Diabetes Among Hypertensive Patients

**Figure 4:**
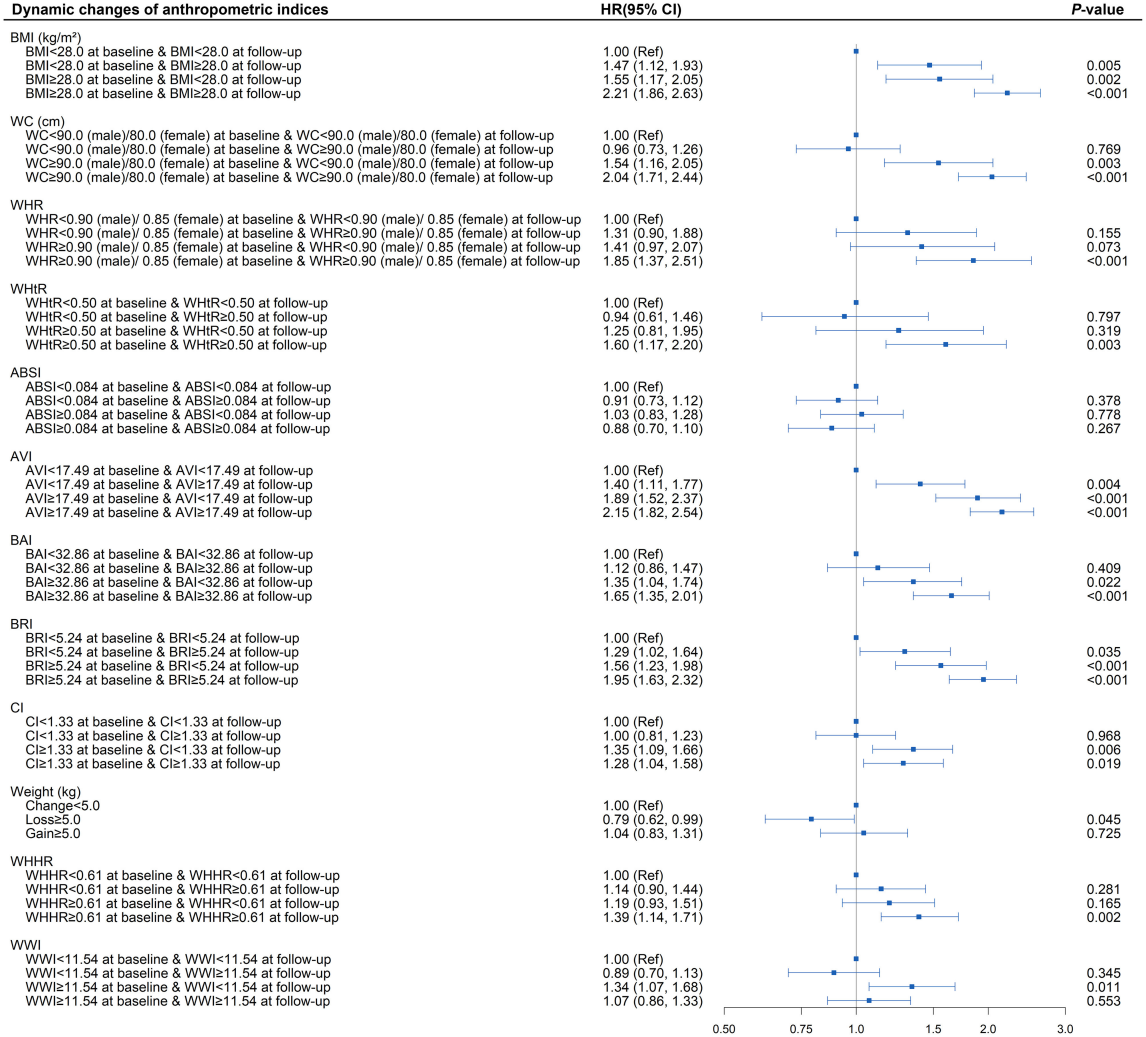
Association between dynamic changes of separate anthropometric indices with diabetes (body mass index [BMI], waist circumference [WC], waist-to-hip ratio [WHR], waist-to-height ratio [WHtR], a body shape index [ABSI], abdominal volume index [AVI], body adiposity index [BAI], body roundness index [BRI], conicity index [CI], weight, waist-hip-height ratio [WHHR], weight-adjusted-waist index [WWI]). The correlation was assessed by multivariate cox regression analysis, adjusted by sex, age, smoking status, drinking status, fasting plasma glucose, serum triglycerides, total cholesterol, high-density lipoprotein, low-density lipoprotein, systolic blood pressure, and diastolic blood pressure at baseline. Hazard ratios (HRs) of the anthropometric indices were represented as the squares and 95% confidence intervals (CIs) by the lines through the squares.

As shown in Supplementary Table 8 and Figure 4, in the fully adjusted model, elevated BRI (BRI > 5.24) was associated with a higher risk of developing diabetes (*P* < 0.05) compared with the subjects whose BRI was <5.24 at baseline and follow-up. Diabetes risk increased significantly when patients with baseline BRI <5.24 progressed to more than 5.24 during the follow-up (HR = 1.29; 95% CI, 1.02, 1.64; *P* = 0.035). There was also a decreasing trend toward diabetes risk when baseline BRI more than 5.24 reversed to <5.24 at follow-up (HR = 1.56; 95% CI, 1.23, 1.98; *P* < 0.001) compared with those whose BRI remained more than 5.24 at follow-up (HR = 1.95; 95% CI, 1.63, 2.32; *P* < 0.001). The highest risk of diabetes onset was observed when BRI was more than 5.24 both at baseline and follow-up. Similar patterns were also observed in BMI and AVI. BRI and AVI, as indicators of central obesity, were sensitive to diabetes risk and were capable of reflecting the risk condition of the patient on diabetes onset.

### Predictive Capabilities of Various Anthropometric Indices for Diabetes Among Hypertensive Patients

**Figure 5:**
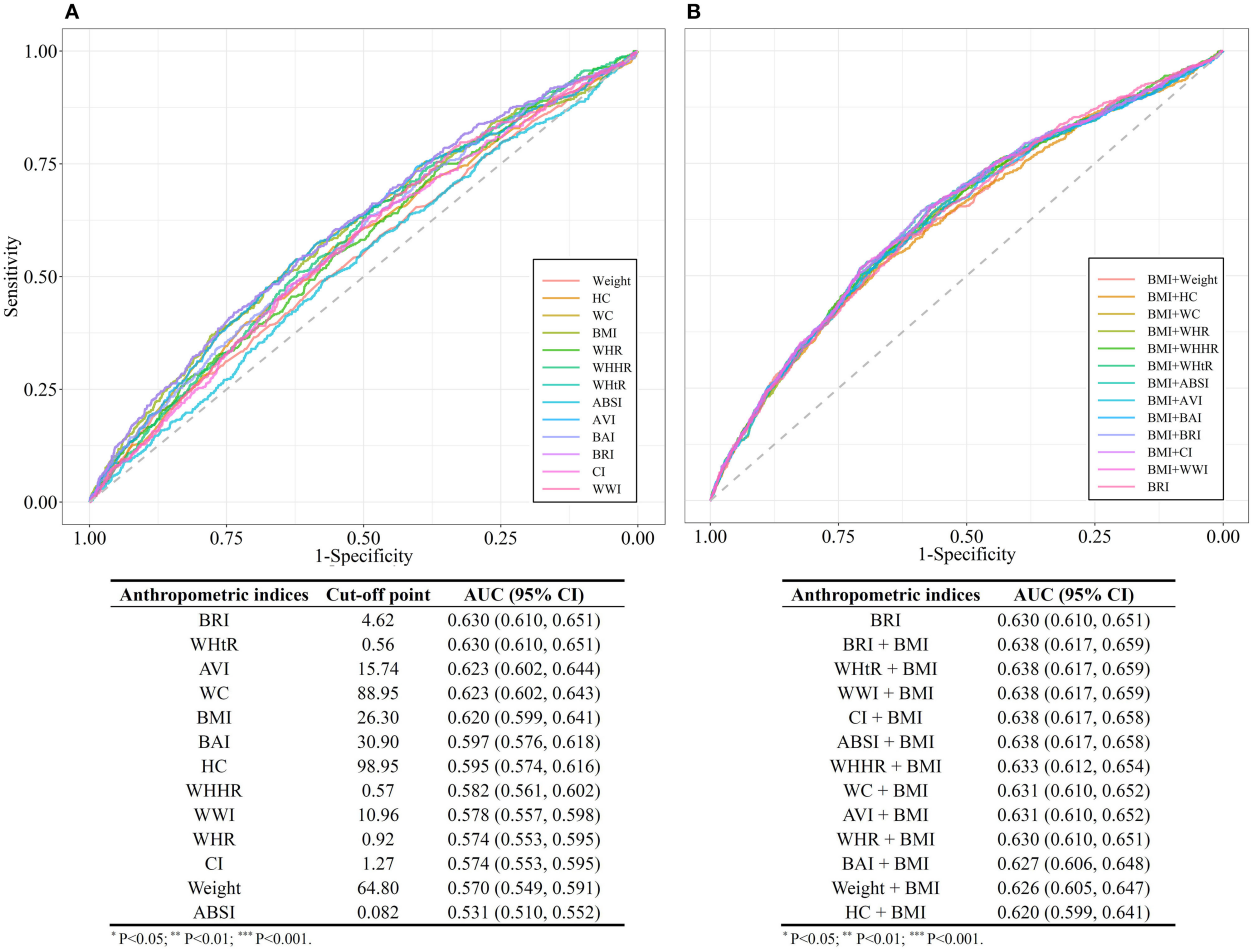
**(A)** Comparison of the receiver operating characteristic (ROC) curves for separate anthropometric indices (body mass index [BMI], waist circumference [WC], waist-to-hip ratio [WHR], waist-to-height ratio [WHtR], a body shape index [ABSI], abdominal volume index [AVI], body adiposity index [BAI], body roundness index [BRI], conicity index [CI], hip circumference [HC], weight, waist-hip-height ratio [WHHR], weight-adjusted-waist index [WWI]). **(B)** Comparison of the receiver operating characteristic (ROC) curves for the combinations of body mass index (BMI) and other anthropometric indices (Weight, WC, HC, WHR, WHtR, ABSI, AVI, BAI, BRI, CI, WHHR, and WWI).

The area under the ROC curve (AUC) was calculated to evaluate the capabilities of each anthropometric measure for the predicting of diabetes among hypertensive patients. As outlined in Figure 5, the AUC values of all the anthropometric indices ranged from 0.50 to 0.70, suggesting a moderate predictive significance for diabetes among hypertensive patients. BRI and WHtR exhibited the largest AUCs for predicting diabetes onset risk (both AUC = 0.63; 95% CI, 0.61, 0.65) among these anthropometric measures. The optimal cut-off value of BRI was determined at 4.62 among the overall hypertensive population, with 3.86 for men, 4.01 for pre-menopausal women, and 5.08 for post-menopausal women (Supplementary Table 9).

We further compared the AUCs of different models constituted by indicator for general obesity BMI and each of the indicators for central obesity. The models combined BMI with BRI or WHtR or WWI (AUC = 0.64; 95% CI, 0.62, 0.66), had a better predictive performance compared with BRI alone.

## Discussion

Individuals with both hypertension and diabetes have been confirmed to significantly increase the risk of CVD morbidity and mortality compared with those with either condition alone. Several anthropometric indices have been shown well to predict the progression of diabetes among general populations. However, less is known on the capabilities of the anthropometric indices in predicting the risk of diabetes among hypertensive patients. Hence, this study aimed to evaluate the potential of different anthropometric indices for predicting diabetes risk among hypertensive patients.

In this cohort study among hypertensive patients with the maximum follow-up of 6 years, the elevated overall and abdominal obesity indicators we examined were positively associated with the increased incident risk of diabetes. Among them, BRI, a novel central obesity index estimated with the use of height and WC whose baseline value and dynamic changes both sensitively reflect the occurrence and progression of diabetes among patients with hypertension, appeared to be the most superior predictor and independent determinant for incident diabetes in the hypertensive population. More importantly, hypertensive patients with elevated BRI, regardless of overall obesity status, were both at higher risk of diabetes. Our study indicated measuring measurements of central obesity, especially the BRI, in addition to BMI, could help to identify patients at a high risk of diabetes among the hypertensive population early.

Hypertensive individuals with a BMI over 25 kg/m^2^ (23 kg/m^2^ for Asian Americans) are recommended to undergo a test of risk for future diabetes according to the American Diabetes Association (30). Nevertheless, currently, the concept of diagnosing obesity using BMI has been challenged. Our findings indicated that BRI and WHtR should be considered the best anthropometric indices in predicting diabetes risk, which exhibited similar predictabilities (AUC = 0.63) in identify diabetes risk and slightly surpassed the performance of BMI (AUC = 0.62). Similarly, prior cross-sectional research on this topic and the literature among the general population with the outcome of diabetes all showed a slight increase in AUC values for WHtR compared with BMI. The slight differences observed could be due to the insensitivity of AUC to the model improvement, performing as a small incremental change when adding a critical risk factor to the model (31). Even so, our results and the mentioned above studies all persuasively supported that some anthropometric measures of central obesity, such as WHtR and BRI, were more robust predictors of diabetes than BMI (14). It is also noteworthy that BRI and WHtR showed similar predictive capability for diabetes among hypertensive patients, which is possibly due to the reason that BRI is a one-to-one non-linear transformation of the WHtR, both based on WC and height. BMI is a measure of both fat and fat-free mass, while WC is an indicator for abdominal fat accumulation more closely correlated with insulin resistance than BMI (32), which might explain why BRI and WHtR could have better performances than BMI in predicting diabetes. From the initial analyses of the present study, although BRI and WHtR had the same AUC, it would seem that BRI was better than WHtR based on the HR values on the association between the dynamic changes in indices and diabetes risk; Among hypertensive patients, while those with BRI elevated during the follow-up was associated with a higher risk of developing diabetes, which was not found in patients with elevated WHtR during follow-up. The strength of the BRI over the WHtR is that the distribution of values of BRI could also be applied to estimate the body fat percentage and thus better reflect the physical health conditions. In addition, lower levels of WHtR and BRI during follow-up were both found to have a tendency toward association with decreased risk of diabetes, although the differences did not achieve statistical significance, which may have been because of the relatively short follow-up duration. Therefore, through long-term monitoring of these simple and non-invasive anthropometric measures and timely intervention, such as regular exercise, dietary control, and weight control, it was expected to promote a shift from abnormal toward normal levels of these indices, which was essential for the prevention of diabetes.

Epidemiologically, the prevalence of obesity in Asians is lower than the Caucasians, yet Asian populations are more easily susceptible to diabetes despite relatively low BMI. This could potentially be attributed to the fact that in general, obesity is defined by BMI, which does not consider central obesity in the clinical guidelines. Thus, people with normal BMI and central obesity are usually neglected (33). This viewpoint has been supported in our research. In this study, hypertensive patients with central obesity defined by WC, WHR, and WHtR had a significantly elevated incident risk of diabetes even in the absence of general obesity. There appear to be very few studies focusing on the diabetes incident risk among hypertensive patients with central obesity. In analogy to our findings, a cross-sectional study showed that central obesity including WC and WHtR were both independently related to pre-diabetes or diabetes after adjusting for BMI among the Asian hypertensive population (34). The potential mechanism of central obesity in promoting diabetes development could be via the role of abdominal fat as a marker of excess ectopic fat, which is key to metabolic abnormality and future development of diabetes (35, 36). In addition, the abdominal fat has more metabolically activity than subcutaneous fat, secreting a variety of lipoxins that have adverse effects on the body, and thus leads to hyperinsulinemia, increasing insulin resistance and enhancing inflammatory responses, which are established determinants of diabetes (37). Further, gratifyingly, BRI also performed similarly in reflecting the central obesity as WC, WHR, and WHtR in this study, showing a satisfactory identification ability of abdominal obesity. Equally important is that the combination of BMI and several anthropometric measures of central obesity could significantly increase predictive power than using a single index. Based on the above information, BMI should be used in conjunction with anthropometric measures of central obesity, of which BRI is a viable choice with a good performance.

BRI was a novel anthropometric index first developed by Thomas et al. (19), used for predicting the percentage of body fat, visceral fat, and provide an initial impression of physical health status. Up to date, BRI has been applied to predict metabolic syndrome in the general population, overweight and obese population, diabetic population, post-menopausal women, and all showed relatively good predictive performances (38, 39). BRI was also considered to be strongly correlated with diabetes and capable of identifying diabetes according to the previous cross-sectional studies (40, 41). The prior cohort study among the elderly population showed that BRI had a certain predictive capacity for diabetes (AUC: 0.609–0.629) (42), which was consistent with our result. Cut-off values of BRI ranged from 3.18 to 6.20 among different studies (42–46), which could be due to the differences in study populations, race, and diagnostic criteria. In the present study, the cut-off point for BRI was 4.62 among the overall hypertensive population, 3.86 in men, 4.01 in pre-menopausal women, and 5.08 in post-menopausal women, which were all within the range of BRI from the previous studies. No significant variation was detected in the cut-off points of BRI between men and pre-menopausal women, yet there was a difference between post-menopausal women and pre-menopausal women/men. This could be explained as prolonged estrogen deficiency among post-menopausal women. Estrogen regulates fat distribution and adipocyte differentiation, thus increasing the risk of weight gain and obesity in postmenopausal women, especially central obesity, suggesting that the optimum cut points for BRI should be selected based on gender and menstrual status of women. Therefore, hypertensive patients with BRI more than the corresponding cut-off value, regardless of the general obesity status, were at a high risk of diabetes, and thus, timely blood glucose monitoring and effective integrative intervention should be conducted for these patients.

The current study has important implications for clinical practice and public health. To begin with, BRI and WHtR were proposed to be the best indices in predicting diabetes among hypertensive in the present study, both of which could be simply calculated based on WC and height. However, WC is not routinely obtained in most clinical settings in China. Indeed, WC measurement could be easily implemented in different levels of the hospitals with only a tape used and simple standardized training of the healthcare personnel. Therefore, BRI could emerge as the screening instrument to remind the healthcare professionals of the hypertensive patients at high risk of diabetes, providing additional benefits beyond BMI measurement. From the point of view of public health, using BRI as a non-invasive, simple predicting tool could help reduce the number of patients required for blood sampling to some degree and offer a practical approach of screening diabetes risk, especially for patients in areas with relatively poor medical resources. In addition, since comorbidities between diabetes and hypertension significantly increase the risk of CVD (5), applying a diabetes risk prediction tool among hypertensive patients appears beneficial for clinicians to better develop intervention strategies, leading to better prevention of CVD. More importantly, the dynamic changes of BRI could sensitively reflect the variation of diabetes onset risk. Since the height remained nearly unchanged, our findings emphasized that the decrease in WC is critical for public health preventive interventions for diabetes. For the above reasons, we thus recommend BRI as a pre-screening tool for diabetes and as a risk stratification tool for CVD among Chinese hypertensive patients.

The following limitations should be considered when interpreting our findings. First, the AUC values of all anthropometric measures in this study were <0.7, which implied modest discrimination performance. Secondly, this was a single-center study. Though Dongguan City is a very representative medium-developed urbanized rural area in China, considering the difference in lifestyles among the regions, our study might not represent the whole population. Third, some factors known to be associated with further development of diabetes, such as family history, dietary habits, and physical activity status, have not been accounted for in the present study. Fourth, the present study did not take hypertension-related target organ damage, such as left ventricular hypertrophy and carotid atherosclerosis, and the duration of hypertension into account, which was crucial to understanding the hypertensive status. Finally, due to a lack of uniform criteria for the novel anthropometric indices in the Chines population, the 75% value was initially selected as the cut-off point to explore the association with diabetes risk in this study. Therefore, further studies with a larger sample size from a multicenter population and a more rigorous experimental design were needed to validate our findings.

In conclusion, our results showed that anthropometric indices for both general obesity and central obesity studied in this study were closely associated with the diabetes onset risk among hypertensive patients. Compared with other anthropometric indices, BRI tends to perform optimally in predicting diabetes among the hypertensive population due to the superior sensitivity of both its baseline value and dynamic changes on reflecting the development of diabetes. Anthropometric indices for central obesity, especially BRI, could be measured in addition to the BMI, which could provide additional clinical and public health benefits in the pre-screening of hypertensive patients with a high risk of diabetes. Furthermore, our findings suggested that hypertensive patients with a BRI of more than 4.62, regardless of the general obesity status, are considered to be at high risk of diabetes. Therefore, interventions focusing on reducing WC were recommended being timely carried out among these patients to reduce the risk of diabetes.

## Data Availability Statement

The raw data supporting the conclusions of this article will be made available by the authors, upon reasonable request.

## Ethics Statement

The present study was approved by the Medical Research Ethics Committee of Guangdong Provincial People's Hospital. All participants provided their written informed consent to participate in this study.

## Author Contributions

JK, YF, and HG: conceptualization. HG, JK, YF, ST, and HC: methodology. YF and JK: validation, writing—review, editing, and supervision. YL and XL: formal analysis. XF: investigation. QZ: resources. SZ: data curation. YL: writing—original draft preparation. XL: visualization. JK, YF, and ST: project administration. JK and HC: funding acquisition. All authors have contributed to the creation of this manuscript for important intellectual content, contributed to the article, and approved the submitted version.

## Funding

This work was supported by the National Key R&D Program of China under Grant No. 2018YFC1314100, the Key-Area Research and Development Program of Guangdong Province under Grant No. 2019B020230001, and the Science and Technology Plan of Guangzhou under Grant No. 201707010330.

## Conflict of Interest

The authors declare that the research was conducted in the absence of any commercial or financial relationships that could be construed as a potential conflict of interest.

## Publisher's Note

All claims expressed in this article are solely those of the authors and do not necessarily represent those of their affiliated organizations, or those of the publisher, the editors and the reviewers. Any product that may be evaluated in this article, or claim that may be made by its manufacturer, is not guaranteed or endorsed by the publisher.
