# Supplementary material for: Body Roundness Index Is a Superior Obesity Index in Predicting Diabetes Risk Among Hypertensive Patients: A Prospective Cohort Study in China
Source: Front Cardiovasc Med. 2021 Nov 18;8:736073. doi: 10.3389/fcvm.2021.736073 (PMC8638826; doi:10.3389/fcvm.2021.736073)
Supplement: Supplementary file 1 [file Data_Sheet_1.docx]

Supplementary Material

# Supplementary Figures and Tables

**Table 1** Formulas of anthropometric indices.

| **Anthropometric indices** | **Formula** |
| --- | --- |
| Body mass index (BMI) | $BMI={Weight}_{(kg)} \div{{Height}^{2}}_{(m)}$ |
| Waist-to-hip ratio (WHR) | $WHR={WC}_{(m)} \div{HC}_{(m)}$ |
| waist-to-height ratio (WHtR) | $WHtR={WC}_{(m)} \div{Height}_{(m)}$ |
| A body shape index (ABSI) | $ABSI={WC}_{(m)} \div({BMI}^{\frac{2}{3}} \times{{Height}^{\frac{1}{2}}}_{(m)})$ |
| Abdominal volume index (AVI) | $AVI=\frac{2\times{{WC}_{(cm)}}^{2}+0.7\times{({WC}_{\left( cm \right)}-{HC}_{(cm)})}^{2}}{1000}$ |
| Body adiposity index (BAI) | $BAI={HC}_{\left( cm \right)} \div{{Height}^{1.5}}_{\left( m \right)}-18$ |
| Body roundness index (BRI) | $BRI=364.2- 365.5\times\sqrt{1-{（\frac{{WC}_{(m)}}{2\pi}）}^{2}\div{(0.5\times{Height}_{(m)})}^{2}}$ |
| Conicity index (CI) | $CI=\frac{{WC}_{\left( m \right)}}{0.109\times\sqrt{\frac{{Weight}_{\left( kg \right)}}{{Height}_{\left( m \right)}}}}$ |
| Weight-adjusted-waist-index (WWI) | $WWI={WC}_{(cm)} \div{{Weight}^{\frac{1}{2}}}_{(kg)}$ |
| Waist-hip-height ratio (WHHR) | $WHHR={WC}_{(m)} \div{Height}_{(m)}\div{HC}_{(m)}$ |

Abbreviations: BMI, body mass index; WC, waist circumference; HC, hip circumference; WHtR, waist-to-height ratio; WHR, waist-to-hip ratio; ABSI, A body shape index; AVI, abdominal volume index; BAI, body adiposity index; BRI, body roundness index; CI, conicity index; WWI, weight-adjusted-waist index; WHHR, waist-hip-height ratio.

**Table 2** Distribution characteristics of anthropometric indices among participants studied.

| **Indices** | **Sex** | **25%** | **50%** | **75%** | **90%** | **95%** |
| --- | --- | --- | --- | --- | --- | --- |
| Weight (kg) | Male | 60.5 | 68 | 75 | 82.4 | 87 |
|  | Female | 51 | 58 | 64.95 | 71 | 76 |
|  | Total | 55 | 62.5 | 71 | 78.3 | 83.45 |
| BMI (kg/m^2^) | Male | 22.95 | 25.01 | 27.22 | 29.38 | 30.92 |
|  | Female | 22.52 | 24.78 | 27.34 | 29.94 | 31.63 |
|  | Total | 22.77 | 24.91 | 27.29 | 29.59 | 31.22 |
| WC (cm) | Male | 82.5 | 88 | 94 | 99 | 102.44 |
|  | Female | 80 | 86 | 93 | 98.08 | 102 |
|  | Total | 81.2 | 87 | 93.27 | 99 | 102 |
| HC (cm) | Male | 91 | 95 | 100 | 104 | 107.2 |
|  | Female | 90 | 95 | 100 | 105 | 107.8 |
|  | Total | 90 | 95 | 100 | 104.5 | 107.5 |
| WHtR | Male | 0.5 | 0.54 | 0.57 | 0.6 | 0.62 |
|  | Female | 0.53 | 0.57 | 0.61 | 0.65 | 0.68 |
|  | Total | 0.51 | 0.55 | 0.59 | 0.63 | 0.66 |
| WHR | Male | 0.89 | 0.92 | 0.96 | 0.99 | 1.01 |
|  | Female | 0.87 | 0.91 | 0.95 | 0.99 | 1.02 |
|  | Total | 0.88 | 0.92 | 0.96 | 0.99 | 1.01 |
| ABSI | Male | 0.078 | 0.08 | 0.083 | 0.086 | 0.088 |
|  | Female | 0.078 | 0.082 | 0.086 | 0.091 | 0.094 |
|  | Total | 0.078 | 0.081 | 0.084 | 0.089 | 0.092 |
| AVI | Male | 13.67 | 15.56 | 17.71 | 19.61 | 20.99 |
|  | Female | 12.97 | 14.92 | 17.3 | 19.36 | 20.88 |
|  | Total | 13.3 | 15.26 | 17.49 | 19.6 | 20.89 |
| BAI | Male | 25.01 | 27.16 | 29.29 | 31.44 | 32.95 |
|  | Female | 29.64 | 32.3 | 35.37 | 38.54 | 40.28 |
|  | Total | 26.59 | 29.48 | 32.86 | 36.4 | 38.7 |
| BRI | Male | 3.41 | 4.05 | 4.74 | 5.44 | 5.94 |
|  | Female | 3.87 | 4.71 | 5.66 | 6.62 | 7.31 |
|  | Total | 3.59 | 4.33 | 5.24 | 6.13 | 6.79 |
| CI | Male | 1.21 | 1.26 | 1.31 | 1.35 | 1.38 |
|  | Female | 1.23 | 1.28 | 1.35 | 1.42 | 1.46 |
|  | Total | 1.22 | 1.27 | 1.33 | 1.39 | 1.43 |
| WWI | Male | 10.29 | 10.71 | 11.09 | 11.52 | 11.76 |
|  | Female | 10.75 | 11.32 | 12 | 12.71 | 13.12 |
|  | Total | 10.47 | 10.96 | 11.54 | 12.26 | 12.74 |
| WHHR | Male | 0.54 | 0.56 | 0.59 | 0.61 | 0.63 |
|  | Female | 0.56 | 0.6 | 0.63 | 0.67 | 0.7 |
|  | Total | 0.55 | 0.58 | 0.61 | 0.65 | 0.68 |

Abbreviations: BMI, body mass index; WC, waist circumference; HC, hip circumference; WHtR, waist-to-height ratio; WHR, waist-to-hip ratio; ABSI, A body shape index; AVI, abdominal volume index; BAI, body adiposity index; BRI, body roundness index; CI, conicity index; WWI, weight-adjusted-waist index; WHHR, waist-hip-height ratio.

**Table 3** Baseline characteristics between subjects with and without diabetes by sex.

|  | **Male** | | | | **Female** | | | | ***P*-value for male vs female** |
| --- | --- | --- | --- | --- | --- | --- | --- | --- | --- |
|  | **Total** | **Diabetes** | **Non-diabetes** | ***P*-value** | **Total** | **Diabetes** | **Non-diabetes** | ***P*-value** |  |
| n | 3467 | 357 | 3110 |  | 3523 | 459 | 3064 |  |  |
| Age (years) | 56.63 ± 14.11 | 59.26 ± 14.49 | 56.33 ± 14.04 | <0.001^***^ | 61.36 ± 13.35 | 62.56 ± 12.70 | 61.18 ± 13.44 | 0.039 | <0.001^***^ |
| FPG (mmol/L) | 4.85 ± 0.64 | 5.33 ± 0.76 | 4.79 ± 0.60 | <0.001^***^ | 4.94 ± 0.63 | 5.44 ± 0.71 | 4.86 ± 0.58 | <0.001 | <0.001^***^ |
| TG (mmol/L) | 1.53 (1.08-2.29) | 1.80 (1.25-2.80) | 1.50 (1.06-2.23) | <0.001^***^ | 1.52 (1.10-2.15) | 1.87 (1.35-2.65) | 1.47 (1.07-2.08) | <0.001 | 0.001^**^ |
| TC (mmol/L) | 5.03 ± 1.11 | 5.00 ± 1.16 | 5.03 ± 1.10 | 0.660 | 5.26 ± 1.12 | 5.31 ± 1.16 | 5.25 ± 1.11 | 0.265 | <0.001^***^ |
| HDL (mmol/L) | 1.24 ± 0.36 | 1.22 ± 0.43 | 1.25 ± 0.35 | 0.213 | 1.37 ± 0.34 | 1.36 ± 0.38 | 1.38 ± 0.33 | 0.217 | <0.001^***^ |
| LDL (mmol/L) | 2.83 ± 0.81 | 2.85 ± 0.91 | 2.82 ± 0.80 | 0.541 | 2.87 ± 0.81 | 2.91 ± 0.80 | 2.86 ± 0.81 | 0.287 | 0.029^*^ |
| UA (μmol/L) | 424.10 ± 107.17 | 437.03 ± 112.26 | 422.62 ± 106.49 | 0.016^*^ | 354.18 ± 96.44 | 375.87 ± 98.27 | 350.96 ± 95.77 | <0.001 | <0.001^***^ |
| Scr (μmol/L) | 88.55 ± 34.32 | 89.01 ± 28.68 | 88.50 ± 34.91 | 0.790 | 66.81 ± 30.12 | 67.01 ± 20.51 | 66.78 ± 31.31 | 0.881 | <0.001^***^ |
| eGFR (mL/(min·1.73 m²)) | 86.57 ± 23.13 | 84.66 ± 23.21 | 86.79 ± 23.11 | 0.101 | 85.33 ± 20.47 | 83.58 ± 19.95 | 85.60 ± 20.54 | 0.05 | 0.018^*^ |
| UAER (mg/24h) | 21.50 (9.70-62.62) | 26.00 (14.30-109.30) | 21.00 (9.57-59.80) | <0.001^***^ | 25.00 (12.30-79.41) | 43.95 (18.58-142.52) | 24.30 (11.80-74.00) | <0.001 | <0.001^***^ |
| Weight (kg) | 68.32 ± 11.24 | 70.68 ± 12.75 | 68.05 ± 11.03 | <0.001^***^ | 58.17 ± 10.37 | 62.09 ± 11.04 | 57.59 ± 10.14 | <0.001 | <0.001^***^ |
| BMI (kg/m²) | 25.12 ± 3.47 | 26.11 ± 3.97 | 25.01 ± 3.39 | <0.001^***^ | 25.07 ± 3.81 | 26.75 ± 4.08 | 24.82 ± 3.71 | <0.001 | 0.546 |
| WC (cm) | 88.19 ± 8.99 | 90.99 ± 9.65 | 87.87 ± 8.85 | <0.001^***^ | 86.51 ± 9.31 | 90.50 ± 9.49 | 85.91 ± 9.13 | <0.001 | <0.001^***^ |
| HC (cm) | 95.46 ± 7.37 | 96.70 ± 8.78 | 95.32 ± 7.18 | 0.001^**^ | 94.88 ± 7.98 | 97.75 ± 8.40 | 94.45 ± 7.82 | <0.001 | 0.002^**^ |
| WHtR | 0.54 ± 0.05 | 0.55 ± 0.06 | 0.53 ± 0.05 | <0.001^***^ | 0.57 ± 0.06 | 0.60 ± 0.07 | 0.57 ± 0.06 | <0.001 | <0.001^***^ |
| WHR | 0.92 ± 0.06 | 0.94 ± 0.10 | 0.92 ± 0.05 | <0.001^***^ | 0.91 ± 0.07 | 0.93 ± 0.07 | 0.91 ± 0.07 | <0.001 | <0.001^***^ |
| ABSI | 0.080 (0.005) | 0.080 (0.005) | 0.081 (0.005) | 0.006^**^ | 0.082 (0.007) | 0.082 (0.007) | 0.082 (0.006) | 0.611 | <0.001^***^ |
| AVI | 15.77 ± 3.16 | 16.79 ± 3.53 | 15.66 ± 3.10 | <0.001^***^ | 15.22 ± 3.21 | 16.63 ± 3.38 | 15.01 ± 3.13 | <0.001 | <0.001^***^ |
| BAI | 27.22 ± 3.51 | 27.99 ± 4.12 | 27.13 ± 3.42 | <0.001^***^ | 32.67 ± 4.61 | 34.19 ± 4.87 | 32.44 ± 4.53 | <0.001 | <0.001^***^ |
| BRI | 4.11 ± 1.08 | 4.49 ± 1.20 | 4.06 ± 1.05 | <0.001^***^ | 4.83 ± 1.39 | 5.41 ± 1.47 | 4.75 ± 1.36 | <0.001 | <0.001^***^ |
| CI | 1.26 ± 0.07 | 1.28 ± 0.07 | 1.26 ± 0.07 | <0.001^***^ | 1.29 ± 0.10 | 1.31 ± 0.09 | 1.29 ± 0.10 | <0.001 | <0.001^***^ |
| WWI | 10.71 ± 0.66 | 10.87 ± 0.67 | 10.69 ± 0.65 | <0.001^***^ | 11.41 ± 0.96 | 11.55 ± 0.92 | 11.39 ± 0.96 | 0.001 | <0.001^***^ |
| WHHR | 0.56 ± 0.04 | 0.58 ± 0.07 | 0.56 ± 0.04 | <0.001^***^ | 0.60 ± 0.06 | 0.60 ± 0.06 | 0.61 ± 0.06 | <0.001 | <0.001^***^ |
| SBP (mmHg) | 138.25 ± 16.78 | 140.88 ± 17.62 | 137.95 ± 16.66 | 0.002^**^ | 140.23 ± 17.26 | 141.76 ± 17.53 | 140.00 ± 17.21 | 0.041 | <0.001^***^ |
| DBP (mmHg) | 86.79 ± 11.44 | 86.33 ± 12.36 | 86.84 ± 11.33 | 0.428 | 83.83 ± 10.56 | 84.19 ± 11.00 | 83.77 ± 10.49 | 0.435 | <0.001^***^ |
| Smoking (n (%)) | 1680 ± 48.5 | 187 ± 52.5 | 1493 ± 48.0 | 0.106 | 18 ± 0.5 | 1 ± 0.2 | 17 ± 0.6 | 0.345 | <0.001^***^ |
| Drinking (n (%)) | 854 ± 24.6 | 81 ± 22.8 | 773 ± 24.9 | 0.383 | 31 ± 0.9 | 2 ± 0.4 | 29 ± 0.9 | 0.275 | <0.001^***^ |

Continuous data are shown as the mean ± SD or median (Q1-Q3), and categorical data as n (%).

Abbreviations: FPG, fasting plasma glucose; TG, triglycerides; TC, total cholesterol; HDL, high-density lipoprotein; LDL, low-density lipoprotein; UA, urid acid; Scr, serum creatinine; eGFR, estimated glomerular filtration rate; BMI, body mass index; WC, waist circumference; HC, hip circumference; WHtR, waist-to-height ratio; WHR, waist-to-hip ratio; ABSI, A body shape index ; AVI, abdominal volume index; BAI, body adiposity index; BRI, body roundness index; CI, conicity index; WWI, weight-adjusted-waist index; WHHR, waist-hip-height ratio; SBP, systolic blood pressure; DBP, diastolic blood pressure.

^*^ P-value < 0.05; ^**^ P-value < 0.01; ^***^ P-value < 0.001.

**Table 4** Univariate cox regression models evaluating the association of demographic, biochemical and clinical characteristics, and anthropometric indexes with diabetes.

|  | **HR, 95% CI**  **(Per SD increment for continuous variables)** | ***P*-value** |
| --- | --- | --- |
| Age (years) | 1.08 (1.00, 1.16) | 0.038^*^ |
| Male | 0.93 (0.81, 1.07) | 0.327 |
| TG (mmol/L) | 1.21 (1.16, 1.26) | <0.001^***^ |
| TC (mmol/L) | 1.10 (1.03, 1.18) | 0.007^**^ |
| HDL (mmol/L) | 0.95 (0.88, 1.02) | 0.167 |
| LDL (mmol/L) | 1.08 (1.01, 1.16) | 0.030^*^ |
| UA (μmol/L) | 1.19 (1.11, 1.27) | <0.001^***^ |
| Scr (μmol/L) | 0.99 (0.92, 1.08) | 0.898 |
| eGFR (mL/(min·1.73 m²)) | 0.96 (0.89, 1.02) | 0.196 |
| UAER (mg/24h) | 1.12 (1.05, 1.19) | <0.001^***^ |
| Weight (kg) | 1.31 (1.22, 1.39) | <0.001^***^ |
| BMI (kg/m²) | 1.39 (1.31, 1.48) | <0.001^***^ |
| WC (cm) | 1.47 (1.38, 1.57) | <0.001^***^ |
| HC (cm) | 1.30 (1.22, 1.38) | <0.001^***^ |
| WHtR | 1.43 (1.34, 1.52) | <0.001^***^ |
| WHR | 1.19 (1.15, 1.24) | <0.001^***^ |
| ABSI | 1.09 (1.02, 1.16) | 0.015^*^ |
| AVI | 1.44 (1.35, 1.53) | <0.001^***^ |
| BAI | 1.25 (1.18, 1.33) | <0.001^***^ |
| BRI | 1.39 (1.32, 1.48) | <0.001^***^ |
| CI | 1.22 (1.15, 1.31) | <0.001^***^ |
| WWI | 1.19 (1.12, 1.27) | <0.001^***^ |
| WHHR | 1.21 (1.14, 1.27) | <0.001^***^ |
| SBP (mmHg) | 1.00 (0.94, 1.08) | 0.896 |
| DBP (mmHg) | 0.94 (0.88, 1.01) | 0.077 |
| Smoking (n (%)) | 1.05 (0.89, 1.23) | 0.591 |
| Drinking (n (%)) | 1.14 (0.91, 1.43) | 0.255 |

Abbreviations: TG, triglycerides; TC, total cholesterol; HDL, high-density lipoprotein; LDL, low-density lipoprotein; UA, urid acid; Scr, serum creatinine; eGFR, estimated glomerular filtration rate; BMI, body mass index; WC, waist circumference; HC, hip circumference; WHtR, waist-to-height ratio; WHR, waist-to-hip ratio; ABSI, A body shape index ; AVI, abdominal volume index; BAI, body adiposity index; BRI, body roundness index; CI, conicity index; WWI, weight-adjusted-waist index; WHHR, waist-hip-height ratio; SBP, systolic blood pressure; DBP, diastolic blood pressure.

^*^ *P*-value < 0.05; ^**^ *P*-value < 0.01; ^***^ *P*-value < 0.001.

**Table 5** Multivariate cox regression models evaluating the associations of baseline anthropometric indexes with diabetes.

|  | **Unadjusted model** | | **Model 1** | | **Model 2** | |
| --- | --- | --- | --- | --- | --- | --- |
|  | **HR (95%CI)** | **P-value** | **HR (95%CI)** | **P-value** | **HR (95%CI)** | **P-value** |
| **Weight (kg)** |  |  |  |  |  |  |
| As continuous variables (per SD increment) | 1.31 (1.22, 1.39) | <0.001^***^ | 1.56 (1.45, 1.69) | <0.001^***^ | 1.50 (1.38, 1.62) | <0.001^***^ |
| <55 | 1.00(Reference) | - | 1.00 (Reference) | - | 1.00(Reference) | - |
| 55-62.5 | 1.27 (1.02, 1.57) | 0.032^*^ | 1.49 (1.19, 1.85) | <0.001^***^ | 1.40 (1.13, 1.75) | 0.003^**^ |
| 62.5-71 | 1.58 (1.28, 1.95) | <0.001^***^ | 2.09 (1.66, 2.61) | <0.001^***^ | 1.88 (1.49, 2.36) | <0.001^***^ |
| ≥71 | 2.01 (1.64, 2.46) | <0.001^***^ | 3.16 (2.50, 4.01) | <0.001^***^ | 2.80 (2.20, 3.58) | <0.001^***^ |
| **BMI (kg/m²)** |  |  |  |  |  |  |
| As continuous variables (per SD increment) | 1.39 (1.31, 1.48) | <0.001^***^ | 1.46 (1.37, 1.55) | <0.001^***^ | 1.40 (1.31, 1.49) | <0.001^***^ |
| <24.0 | 1.00 (Reference) | - | 1.00 (Reference) | - | 1.00 (Reference) | - |
| 24.0-28.0 | 1.47 (1.24, 1.75) | <0.001^***^ | 1.58 (1.33, 1.88) | <0.001^***^ | 1.46 (1.23, 1.74) | <0.001^***^ |
| ≥28.0 | 2.50 (2.09, 3.00) | <0.001^***^ | 2.78 (2.31, 3.35) | <0.001^***^ | 2.45 (2.03, 2.97) | <0.001^***^ |
| **WC (cm)** |  |  |  |  |  |  |
| As continuous variables (per SD increment) | 1.47 (1.38, 1.57) | <0.001^***^ | 1.48 (1.39, 1.59) | <0.001^***^ | 1.42 (1.33, 1.52) | <0.001^***^ |
| <90 in male or <80 in female | 1.00 (Reference) | - | 1.00 (Reference) | - | 1.00 (Reference) | - |
| ≥90 in male or ≥80 in female | 2.14 (1.85, 2.48) | <0.001^***^ | 2.20 (1.89, 2.55) | <0.001^***^ | 1.98 (1.70, 2.32) | <0.001^***^ |
| **HC (cm)** |  |  |  |  |  |  |
| As continuous variables (per SD increment) | 1.3 (1.22, 1.38) | <0.001^***^ | 1.34 (1.26, 1.42) | <0.001^***^ | 1.30 (1.22, 1.38) | <0.001^***^ |
| <90 | 1.00 (Reference) | - | 1.00 (Reference) | - | 1.00 (Reference) | - |
| 90-95 | 1.29 (1.02, 1.63) | 0.032^*^ | 1.36 (1.07, 1.71) | 0.011^*^ | 1.26 (0.99, 1.59) | 0.057 |
| 95-100 | 1.60 (1.27, 2.00) | <0.001^***^ | 1.72 (1.37, 2.16) | <0.001 | 1.55 (1.23, 1.95) | <0.001^***^ |
| ≥100 | 2.31 (1.87, 2.86) | <0.001^***^ | 2.56 (2.06, 3.18) | <0.001^***^ | 2.27 (1.82, 2.83) | <0.001^***^ |
| **WHtR** |  |  |  |  |  |  |
| As continuous variables (per SD increment) | 1.43 (1.34, 1.52) | <0.001^***^ | 1.46 (1.37, 1.56) | <0.001^***^ | 1.39 (1.30, 1.49) | <0.001^***^ |
| <0.5 | 1.00 (Reference) | - | 1.00 (Reference) | - | 1.00 (Reference) | - |
| ≥0.5 | 1.9 (1.51, 2.39) | <0.001^***^ | 1.90 (1.51, 2.39) | <0.001^***^ | 1.62 (1.28, 2.05) | <0.001^***^ |
| **WHR** |  |  |  |  |  |  |
| As continuous variables (per SD increment) | 1.19 (1.15, 1.24) | <0.001^***^ | 1.20 (1.15, 1.24) | <0.001^***^ | 1.16 (1.12, 1.21) | <0.001^***^ |
| <0.90 in male or <0.85 in female | 1.00 (Reference) | - | 1.00 (Reference) | - | 1.00 (Reference) | - |
| ≥0.90 in male or ≥0.85 in female | 1.76 (1.45, 2.13) | <0.001^***^ | 1.74 (1.43, 2.12) | <0.001^***^ | 1.53 (1.25, 1.87) | <0.001^***^ |
| **ABSI** |  |  |  |  |  |  |
| As continuous variables (per SD increment) | 1.09 (1.02, 1.16) | 0.015^*^ | 1.06 (0.98, 1.14) | 0.125 | 1.04 (0.96, 1.12) | 0.328 |
| <0.078 | 1.00 (Reference) | - | 1.00(Reference) | - | 1.00 (Reference) | - |
| 0.078-0.081 | 1.20 (0.98, 1.47) | 0.083 | 1.18 (0.96, 1.45) | 0.109 | 1.12 (0.91, 1.37) | 0.293 |
| 0.081-0.084 | 1.35 (1.10, 1.64) | 0.003^**^ | 1.30 (1.06, 1.59) | 0.010^*^ | 1.24 (1.02, 1.53) | 0.035^*^ |
| ≥0.084 | 1.29 (1.06, 1.57) | 0.012^*^ | 1.20 (0.97, 1.49) | 0.093 | 1.11 (0.89, 1.38) | 0.340 |
| **AVI** |  |  |  |  |  |  |
| As continuous variables (per SD increment) | 1.44 (1.35, 1.53) | <0.001^***^ | 1.45 (1.37, 1.55) | <0.001^***^ | 1.40 (1.31, 1.49) | <0.001^***^ |
| <13.30 | 1.00 (Reference) | - | 1.00 (Reference) | - | 1.00 (Reference) | - |
| 13.30-15.26 | 1.25 (0.99, 1.59) | 0.061 | 1.28 (1.01, 1.63) | 0.039^*^ | 1.21 (0.95, 1.54) | 0.119 |
| 15.26-17.49 | 1.84 (1.47, 2.29) | <0.001^***^ | 1.87 (1.50, 2.34) | <0.001^***^ | 1.69 (1.35, 2.12) | <0.001^***^ |
| ≥17.49 | 2.88 (2.34, 3.54) | <0.001^***^ | 2.95 (2.40, 3.63) | <0.001^***^ | 2.57 (2.08, 3.18) | <0.001^***^ |
| **BAI** |  |  |  |  |  |  |
| As continuous variables (per SD increment) | 1.25 (1.18, 1.33) | <0.001^***^ | 1.33 (1.24, 1.43) | <0.001^***^ | 1.27 (1.18, 1.37) | <0.001^***^ |
| <26.59 | 1.00 (Reference) | - | 1.00 (Reference) | - | 1.00 (Reference) | - |
| 26.59-29.48 | 1.35 (1.08, 1.68) | 0.009^**^ | 1.42 (1.13, 1.78) | 0.002^**^ | 1.34 (1.07, 1.68) | 0.012^*^ |
| 29.48-32.86 | 1.36 (1.09, 1.69) | 0.006^**^ | 1.57 (1.25, 1.98) | <0.001^***^ | 1.42 (1.12, 1.80) | 0.003^**^ |
| ≥32.86 | 1.91 (1.56, 2.34) | <0.001^***^ | 2.35 (1.84, 2.99) | <0.001^***^ | 1.99 (1.56, 2.55) | <0.001^***^ |
| **BRI** |  |  |  |  |  |  |
| As continuous variables (per SD increment) | 1.39 (1.32, 1.48) | <0.001^***^ | 1.42 (1.34, 1.51) | <0.001^***^ | 1.36 (1.28, 1.45) | <0.001^***^ |
| <3.59 | 1.00 (Reference) | - | 1.00 (Reference) | - | 1.00 (Reference) | - |
| 3.59-4.33 | 1.46 (1.15, 1.85) | 0.002^**^ | 1.46 (1.15, 1.86) | 0.002^**^ | 1.35 (1.06, 1.73) | 0.014^*^ |
| 4.33-5.24 | 1.94 (1.55, 2.44) | <0.001^***^ | 2.00 (1.59, 2.52) | <0.001^***^ | 1.80 (1.43, 2.27) | <0.001^***^ |
| ≥5.24 | 2.77 (2.23, 3.43) | <0.001^***^ | 2.91 (2.33, 3.63) | <0.001^***^ | 2.47 (1.97, 3.11) | <0.001^***^ |
| **CI** |  |  |  |  |  |  |
| As continuous variables (per SD increment) | 1.22 (1.15, 1.31) | <0.001^***^ | 1.22 (1.14, 1.31) | <0.001^***^ | 1.18 (1.10, 1.27) | <0.001^***^ |
| <1.22 | 1.00 (Reference) | - | 1.00 (Reference) | - | 1.00 (Reference) | - |
| 1.22-1.27 | 1.27 (1.01, 1.58) | 0.037^*^ | 1.26 (1.01, 1.58) | 0.039^*^ | 1.16 (0.93, 1.45) | 0.199 |
| 1.27-1.33 | 1.76 (1.43, 2.16) | <0.001^***^ | 1.74 (1.42, 2.15) | <0.001^***^ | 1.56 (1.27, 1.93) | <0.001^***^ |
| ≥1.33 | 1.89 (1.54, 2.32) | <0.001^***^ | 1.87 (1.51, 2.32) | <0.001^***^ | 1.67 (1.34, 2.07) | <0.001^***^ |
| **WWI** |  |  |  |  |  |  |
| As continuous variables (per SD increment) | 1.19 (1.12, 1.27) | <0.001^***^ | 1.22 (1.13, 1.32) | <0.001^***^ | 1.17 (1.08, 1.27) | <0.001^***^ |
| <10.47 | 1.00 (Reference) | - | 1.00 (Reference) | - | 1.00 (Reference) | - |
| 10.47-10.96 | 1.45 (1.16, 1.81) | 0.001^**^ | 1.45 (1.16, 1.82) | 0.001^**^ | 1.37 (1.09, 1.71) | 0.006^**^ |
| 10.96-11.54 | 1.77 (1.43, 2.18) | <0.001^***^ | 1.81 (1.46, 2.26) | <0.001^***^ | 1.63 (1.31, 2.04) | <0.001^***^ |
| ≥11.54 | 1.87 (1.52, 2.30) | <0.001^***^ | 1.99 (1.57, 2.52) | <0.001^***^ | 1.73 (1.35, 2.20) | <0.001^***^ |
| **WHHR** |  |  |  |  |  |  |
| As continuous variables (per SD increment) | 1.21 (1.14, 1.27) | <0.001^***^ | 1.22 (1.15, 1.29) | <0.001^***^ | 1.17 (1.10, 1.24) | <0.001^***^ |
| <0.55 | 1.00 (Reference) | - | 1.00 (Reference) | - | 1.00 (Reference) | - |
| 0.55-0.58 | 1.14 (0.92, 1.42) | 0.236 | 1.16 (0.93, 1.45) | 0.189 | 1.07 (0.85, 1.33) | 0.580 |
| 0.58-0.61 | 1.53 (1.24, 1.88) | <0.001^***^ | 1.57 (1.27, 1.94) | <0.001^***^ | 1.36 (1.10, 1.69) | 0.005^**^ |
| ≥0.61 | 1.68 (1.37, 2.06) | <0.001^***^ | 1.76 (1.41, 2.21) | <0.001^***^ | 1.51 (1.19, 1.90) | <0.001^***^ |

Abbreviations: BMI, body mass index; WC, waist circumference; HC, hip circumference; WHtR, waist-to-height ratio; WHR, waist-to-hip ratio; ABSI, A body shape index; AVI, abdominal volume index; BAI, body adiposity index; BRI, body roundness index; CI, conicity index; WWI, weight-adjusted-waist index; WHHR, waist-hip-height ratio.

Model 1: adjusted by sex, age, smoking status, drinking status.

Model 2: adjusted by model 1 plus TG, TC, HDL, LDL, SBP, DBP.

^*^ *P*-value < 0.05; ^**^ *P* -value < 0.01; ^***^ *P* -value < 0.001.

**Table 6** Multivariate cox regression models evaluating the associations of different combinations of BMI and anthropometric indices of central obesity with diabetes.

|  | **Event/**  **Number** | **Unadjusted model** | | **Model 1** | | **Model 2** | |
| --- | --- | --- | --- | --- | --- | --- | --- |
|  |  | **HR (95%CI)** | ***P*-value** | **HR (95%CI)** | ***P*-value** | **HR (95%CI)** | ***P*-value** |
| **BMI (kg/m^2^) & WC (cm)** |  |  |  |  |  |  |  |
| BMI<28 & WC<90 (male)/80 (female) | 251/3381 | 1.00 | - | 1.00 | - | 1.00 | - |
| BMI≥28 & WC<90 (male)/80 (female) | 6/71 | 0.99 (0.44, 2.23) | 0.981 | 1.09 (0.48, 2.45) | 0.838 | 1.04 (0.46, 2.35) | 0.919 |
| BMI<28 & WC≥90 (male)/80 (female) | 304/2283 | 1.80 (1.52, 2.12) | <0.001^***^ | 1.82 (1.53, 2.15) | <0.001^***^ | 1.68 (1.41, 1.99) | <0.001^***^ |
| BMI≥28 & WC≥90 (male)/80 (female) | 255/1255 | 2.77 (2.33, 3.30) | <0.001^***^ | 2.95 (2.47, 3.53) | <0.001^***^ | 2.63 (2.19, 3.17) | <0.001^***^ |
| **BMI (kg/m^2^) & WHtR** |  |  |  |  |  |  |  |
| BMI<28 & WHtR<0.5 | 82/1225 | 1.00 | - | 1.00 | - | 1.00 | - |
| BMI≥28 & WHtR<0.5 | 0/2 | / | - | - | - | - | - |
| BMI<28 & WHtR≥0.5 | 473/4439 | 1.58 (1.25, 2.00) | <0.001^***^ | 1.56 (1.23, 1.98) | <0.001^***^ | 1.38 (1.09, 1.76) | 0.008^**^ |
| BMI≥28 & WHtR≥0.5 | 261/1324 | 2.95 (2.30, 3.78) | <0.001^***^ | 3.08 (2.39, 3.96) | <0.001^***^ | 2.57 (1.98, 3.33) | <0.001^***^ |
| **BMI (kg/m^2^) & WHR** |  |  |  |  |  |  |  |
| BMI<28 & WHR<0.9 (male)/ 0.85 (female) | 97/1533 | 1.00 | - | 1.00 | - | 1.00 | - |
| BMI≥28 & WHR<0.9 (male)/ 0.85 (female) | 24/107 | 3.82 (2.44, 5.97) | <0.001^***^ | 4.17 (2.66, 6.54) | <0.001^***^ | 3.97 (2.52, 6.25) | <0.001^***^ |
| BMI<28 & WHR≥0.9 (male)/ 0.85 (female) | 458/4131 | 1.76 (1.41, 2.19) | <0.001^***^ | 1.72 (1.38, 2.15) | <0.001^***^ | 1.57 (1.25, 1.96) | <0.001^***^ |
| BMI≥28 & WHR≥0.9 (male)/ 0.85 (female) | 237/1219 | 3.07 (2.43, 3.89) | <0.001^***^ | 3.19 (2.51, 4.05) | <0.001^***^ | 2.71 (2.12, 3.47) | <0.001^***^ |
| **BMI (kg/m^2^) & ABSI** |  |  |  |  |  |  |  |
| BMI<28 & ABSI<0.084 | 374/4109 | 1.00 | - | 1.00 | - | 1.00 | - |
| BMI≥28 & ABSI<0.084 | 220/1133 | 2.15 (1.82, 2.54) | <0.001^***^ | 2.24 (1.89, 2.66) | <0.001^***^ | 2.03 (1.71, 2.42) | <0.001^***^ |
| BMI<28 & ABSI≥0.084 | 181/1555 | 1.26 (1.05, 1.50) | 0.012^*^ | 1.15 (0.95, 1.39) | 0.154 | 1.09 (0.90, 1.32) | 0.358 |
| BMI≥28 & ABSI≥s0.084 | 41/193 | 2.21 (1.60, 3.05) | <0.001^***^ | 2.07 (1.49, 2.88) | <0.001^***^ | 1.82 (1.30, 2.53) | <0.001^***^ |
| **BMI (kg/m^2^) & AVI** |  |  |  |  |  |  |  |
| BMI<28 & AVI<17.5 | 431/4868 | 1.00 | - | 1.00 | - | 1.00 | - |
| BMI≥28 & AVI<17.5 | 58/374 | 1.69 (1.28, 2.22) | <0.001^***^ | 1.80 (1.36, 2.37) | <0.001^***^ | 1.70 (1.28, 2.25) | <0.001^***^ |
| BMI<28 & AVI≥17.5 | 124/796 | 1.91 (1.56, 2.33) | <0.001^***^ | 1.89 (1.54, 2.30) | <0.001^***^ | 1.74 (1.42, 2.13) | <0.001^***^ |
| BMI≥28 & AVI≥17.5 | 203/952 | 2.50 (2.11, 2.95) | <0.001^***^ | 2.60 (2.20, 3.08) | <0.001^***^ | 2.33 (1.96, 2.77) | <0.001^***^ |
| **BMI (kg/m^2^) & BAI** |  |  |  |  |  |  |  |
| BMI<28 & BAI<32.86 | 419/4658 | 1.00 | - | 1.00 | - | 1.00 | - |
| BMI≥28 & BAI<32.86 | 99/583 | 1.96 (1.58, 2.44) | <0.001^***^ | 2.08 (1.66, 2.61) | <0.001^***^ | 1.93 (1.54, 2.43) | <0.001^***^ |
| BMI<28 & BAI≥32.86 | 136/1006 | 1.31 (1.08, 1.59) | 0.006^**^ | 1.29 (1.04, 1.60) | 0.020^*^ | 1.22 (0.98, 1.52) | 0.070 |
| BMI≥28 & BAI≥32.86 | 162/743 | 2.27 (1.89, 2.72) | <0.001^***^ | 2.37 (1.95, 2.88) | <0.001^***^ | 2.10 (1.72, 2.56) | <0.001^***^ |
| **BMI (kg/m^2^) & BRI** |  |  |  |  |  |  |  |
| BMI<28 & BRI<5.24 | 428/4812 | 1.00 | - | 1.00 | - | 1.00 | - |
| BMI≥28 & BRI<5.24 | 64/429 | 1.70 (1.31, 2.21) | <0.001^***^ | 1.82 (1.39, 2.38) | <0.001^***^ | 1.74 (1.33, 2.28) | <0.001^***^ |
| BMI<28 & BRI≥5.24 | 127/852 | 1.56 (1.28, 1.91) | <0.001^***^ | 1.52 (1.23, 1.87) | <0.001^***^ | 1.39 (1.13, 1.72) | 0.002^**^ |
| BMI≥28 & BRI≥5.24 | 197/897 | 2.43 (2.05, 2.87) | <0.001^***^ | 2.49 (2.10, 2.96) | <0.001^***^ | 2.21 (1.85, 2.64) | <0.001^***^ |
| **BMI (kg/m^2^) & CI** |  |  |  |  |  |  |  |
| BMI<28 & CI<1.33 | 387/4307 | 1.00 | - | 1.00 | - | 1.00 | - |
| BMI≥28 & CI<1.33 | 170/935 | 2.03 (1.69, 2.43) | <0.001^***^ | 2.13 (1.77, 2.55) | <0.001^***^ | 1.91 (1.58, 2.31) | <0.001^***^ |
| BMI<28 & CI≥1.33 | 168/1357 | 1.40 (1.16, 1.67) | <0.001^***^ | 1.31 (1.08, 1.59) | 0.006^**^ | 1.25 (1.03, 1.51) | 0.024^*^ |
| BMI≥28 & CI≥1.33 | 91/391 | 2.63 (2.10, 3.31) | <0.001^***^ | 2.59 (2.06, 3.27) | <0.001^***^ | 2.35 (1.86, 2.97) | <0.001^***^ |
| **BMI (kg/m^2^) & WWI** |  |  |  |  |  |  |  |
| BMI<28 & WWI<11.54 | 379/4293 | 1.00 | - | 1.00 | - | 1.00 | - |
| BMI≥28 & WWI<11.54 | 174/949 | 2.12 (1.77, 2.53) | <0.001^***^ | 2.23 (1.85, 2.67) | <0.001^***^ | 2.02 (1.68, 2.44) | <0.001^***^ |
| BMI<28 & WWI≥11.54 | 176/1371 | 1.37 (1.15, 1.64) | <0.001^***^ | 1.29 (1.05, 1.57) | 0.014^*^ | 1.20 (0.98, 1.47) | 0.079 |
| BMI≥28 & WWI≥11.54 | 87/377 | 2.40 (1.90, 3.03) | <0.001^***^ | 2.36 (1.85, 3.02) | <0.001^***^ | 2.07 (1.62, 2.66) | <0.001^***^ |
| **BMI (kg/m^2^) & WHHR** |  |  |  |  |  |  |  |
| BMI<28 & WHHR<0.61 | 390/4359 | 1.00 | - | 1.00 | - | 1.00 | - |
| BMI≥28 & WHHR<0.61 | 161/882 | 2.08 (1.73, 2.49) | <0.001^***^ | 2.19 (1.82, 2.65) | <0.001^***^ | 1.98 (1.63, 2.40) | <0.001^***^ |
| BMI<28 & WHHR≥0.61 | 165/1305 | 1.35 (1.13, 1.62) | 0.001^***^ | 1.27 (1.04, 1.55) | 0.021^*^ | 1.19 (0.97, 1.45) | 0.092 |
| BMI≥28 & WHHR≥0.61 | 100/444 | 2.38 (1.91, 2.97) | <0.001^***^ | 2.35 (1.88, 2.95) | <0.001^***^ | 2.11 (1.67, 2.65) | <0.001^***^ |

Abbreviations: BMI, body mass index; WC, waist circumference; HC, hip circumference; WHtR, waist-to-height ratio; WHR, waist-to-hip ratio; ABSI, A body shape index; AVI, abdominal volume index; BAI, body adiposity index; BRI, body roundness index; CI, conicity index; WWI, weight-adjusted-waist index; WHHR, waist-hip-height ratio.

Model 1: adjusted by sex, age, smoking status, drinking status.

Model 2: adjusted by model 1 plus TG, TC, HDL, LDL, SBP, DBP.

^*^ *P*-value < 0.05; ^**^ *P* -value < 0.01; ^***^ *P* -value < 0.001.

**Table 7** Association between BRI and diabetes according to baseline characteristics.

| **Subgroup** | **HR (95%CI)** | | ***P*-value** | ***P*-value for interaction** |
| --- | --- | --- | --- | --- |
|  | **BRI <5.24** | **BRI ≥5.24** |  |  |
| Age (years) |  |  |  | 0.756 |
| <60 | 1.00 | 1.71 (1.37, 2.12) | <0.001 |  |
| ≥60 | 1.00 | 1.91 (1.54, 2.37) | <0.001 |  |
| Sex (n (%)) |  |  |  | 0.252 |
| Male | 1.00 | 1.91 (1.58, 2.31) | <0.001 |  |
| Female | 1.00 | 1.57 (1.20, 2.05) | <0.001 |  |
| TG (mmol/L) |  |  |  | 0.014^*^ |
| <2.3 | 1.00 | 1.86 (1.53, 2.25) | <0.001 |  |
| ≥2.3 | 1.00 | 1.68 (1.31, 2.16) | <0.001 |  |
| TC (mmol/L) |  |  |  | 0.518 |
| <6.2 | 1.00 | 1.64 (1.38, 1.94) | <0.001 |  |
| ≥6.2 | 1.00 | 2.33 (1.62, 3.34) | <0.001 |  |
| HDL (mmol/L) |  |  |  | 0.711 |
| ≥1.0 | 1.00 | 1.71 (1.15, 2.53) | 0.0074 |  |
| <1.0 | 1.00 | 1.78 (1.51, 2.10) | <0.001 |  |
| LDL (mmol/L) |  |  |  | 0.207 |
| <4.1 | 1.00 | 1.69 (1.45, 1.99) | <0.001 |  |
| ≥4.1 | 1.00 | 2.76 (1.59, 4.79) | <0.001 |  |
| UA (μmol/L) |  |  |  | 0.309 |
| ≤420 | 1.00 | 1.86 (1.52, 2.27) | <0.001 |  |
| >420 | 1.00 | 1.50 (1.18, 1.91) | 0.001 |  |
| eGFR (mL/(min·1.73 m²)) |  |  |  | 0.276 |
| ≥90 | 1.00 | 1.61 (1.27, 2.03) | <0.001 |  |
| 60-90 | 1.00 | 1.69 (1.34, 2.13) | <0.001 |  |
| <60 | 1.00 | 2.49 (1.64, 3.78) | <0.001 |  |
| BMI (kg/m²) |  |  |  | <0.001^***^ |
| <28 | 1.00 | 1.20 (0.75, 1.93) | 0.447 |  |
| ≥28 | 1.00 | 1.21 (0.93, 1.59) | 0.162 |  |
| WC (cm) |  |  |  |  |
| <90 in male or <85 in female | 1.00 | 0.28 (0.04, 2.02) | 0.207 | <0.001^***^ |
| ≥90 in male or ≥85 in female | 1.00 | 1.30 (1.08, 1.55) | 0.004 |  |
| HC (cm) |  |  |  | <0.001^***^ |
| <100 | 1.00 | 1.43 (1.13, 1.81) | 0.003 |  |
| ≥100 | 1.00 | 1.43 (1.11, 1.84) | 0.006 |  |
| WHR |  |  |  | 0.832 |
| <0.90 in male or <0.85 in female | 1.00 | 2.92 (1.44, 5.92) | 0.003 |  |
| ≥0.90 in male or ≥0.85 in female | 1.00 | 1.60 (1.36, 1.87) | <0.001 |  |
| WHtR |  |  |  | 0.002^**\^ |
| <0.5 | 1.00 | - | - |  |
| ≥0.5 | 1.00 | 1.64 (1.41, 1.92) | <0.001 |  |
| ABSI |  |  |  | 0.017^*^ |
| <0.084 | 1.00 | 1.86 (1.55, 2.24) | <0.001 |  |
| ≥0.084 | 1.00 | 1.80 (1.34, 2.40) | <0.001 |  |
| AVI |  |  |  | <0.001^***^ |
| <17.5 | 1.00 | 1.35 (1.04, 1.76) | 0.027 |  |
| ≥17.5 | 1.00 | 1.01 (0.75, 1.37) | 0.947 |  |
| BAI |  |  |  | 0.092 |
| <32.9 | 1.00 | 1.64 (1.30, 2.07) | <0.001 |  |
| ≥32.9 | 1.00 | 1.63 (1.23, 2.17) | <0.001 |  |
| CI |  |  |  | 0.260 |
| <1.3 | 1.00 | 1.76 (1.43, 2.17) | <0.001 |  |
| ≥1.3 | 1.00 | 1.74 (1.29, 2.35) | <0.001 |  |
| WWI |  |  |  | 0.070 |
| <11.5 | 1.00 | 1.95 (1.58, 2.41) | <0.001 |  |
| ≥11.5 | 1.00 | 1.74 (1.29, 2.34) | <0.001 |  |
| WHHR |  |  |  | 0.243 |
| <0.6 | 1.00 | 1.77 (1.44, 2.18) | <0.001 |  |
| ≥0.6 | 1.00 | 1.76 (1.33, 2.34) | <0.001 |  |
| SBP (mmHg) |  |  |  | 0.360 |
| <140 | 1.00 | 1.65 (1.30, 2.09) | <0.001 |  |
| ≥140 | 1.00 | 1.81 (1.49, 2.21) | <0.001 |  |
| DBP (mmHg) |  |  |  | 0.868 |
| <90 | 1.00 | 1.78 (1.46, 2.17) | <0.001 |  |
| ≥90 | 1.00 | 1.70 (1.34, 2.16) | <0.001 |  |
| Smoking (n (%)) |  |  |  | 0.912 |
| No | 1.00 | 1.72 (1.45, 2.04) | <0.001 |  |
| Yes | 1.00 | 1.80 (1.26, 2.57) | 0.001 |  |
| Drinking (n (%)) |  |  |  | 0.611 |
| No | 1.00 | 1.73 (1.48, 2.03) | <0.001 |  |
| Yes | 1.00 | 1.55 (0.90, 2.66) | 0.113 |  |

Abbreviations: FPG, fasting plasma glucose; TG, triglycerides; TC, total cholesterol; HDL, high-density lipoprotein; LDL, low-density lipoprotein; UA, urid acid; Scr, serum creatinine; eGFR, estimated glomerular filtration rate; BMI, body mass index; WC, waist circumference; HC, hip circumference; WHtR, waist-to-height ratio; WHR, waist-to-hip ratio; ABSI, A body shape index ; AVI, abdominal volume index; BAI, body adiposity index; BRI, body roundness index; CI, conicity index; WWI, weight-adjusted-waist index; WHHR, waist-hip-height ratio; SBP, systolic blood pressure; DBP, diastolic blood pressure.

^*^ *P*-value < 0.05; ^**^ *P*-value < 0.01; ^***^ *P*-value < 0.001.

**Table 8** Multivariate cox regression models evaluating the associations of dynamic changes of anthropometric indices with diabetes.

|  | **Unadjusted model** | | **Model 1** | | **Model 2** | |
| --- | --- | --- | --- | --- | --- | --- |
|  | **HR (95%CI)** | **P-value** | **HR (95%CI)** | **P-value** | **HR (95%CI)** | **P-value** |
| **Weight change (kg)** |  |  |  |  |  |  |
| <5 | 1.00 | - | 1.00 | - | 1.00 | - |
| Loss ≥5 | 0.79 (0.63, 1.00) | 0.048^*^ | 0.78 (0.62, 0.98) | 0.034^*^ | 0.79 (0.62, 0.99) | 0.045^*^ |
| Gain ≥5 | 1.00 (0.79, 1.25) | 0.972 | 1.01 (0.81, 1.27) | 0.924 | 1.04 (0.83, 1.31) | 0.725 |
| **Dynamic changes of BMI (kg/m^2^)** |  |  |  |  |  |  |
| BMI<28 at baseline & BMI<28 at follow-up | 1.00 | - | 1.00 | - | 1.00 | - |
| BMI<28 at baseline & BMI≥28 at follow-up | 1.58 (1.21, 2.06) | <0.001^***^ | 1.63 (1.25, 2.13) | <0.001^***^ | 1.47 (1.12, 1.93) | 0.005^**^ |
| BMI≥28 at baseline & BMI<28 at follow-up | 1.65 (1.25, 2.17) | <0.001^***^ | 1.72 (1.30, 2.27) | <0.001^***^ | 1.55 (1.17, 2.05) | 0.002^**^ |
| BMI≥28 at baseline & BMI≥28 at follow-up | 2.27 (1.93, 2.67) | <0.001^***^ | 2.44 (2.06, 2.88) | <0.001^***^ | 2.21 (1.86, 2.63) | <0.001^***^ |
| **Dynamic changes of WC (cm)** |  |  |  |  |  |  |
| WC<90 (male)/80 (female) at baseline & WC<90 (male)/80 (female) at follow-up | 1.00 | - | 1.00 | - | 1.00 | - |
| WC<90 (male)/80 (female) at baseline & WC≥90 (male)/80 (female) at follow-up | 0.96 (0.73, 1.26) | 0.748 | 0.99 (0.76, 1.30) | 0.957 | 0.96 (0.73, 1.26) | 0.769 |
| WC≥90 (male)/80 (female) at baseline & WC<90 (male)/80 (female) at follow-up | 1.61 (1.22, 2.14) | <0.001^***^ | 1.65 (1.24, 2.18) | <0.001^***^ | 1.54 (1.16, 2.05) | 0.003^**^ |
| WC≥90 (male)/80 (female) at baseline & WC≥90 (male)/80 (female) at follow-up | 2.21 (1.86, 2.61) | <0.001^***^ | 2.30 (1.93, 2.73) | <0.001^***^ | 2.04 (1.71, 2.44) | <0.001^***^ |
| **Dynamic changes of WHtR** |  |  |  |  |  |  |
| WHtR<0.5 at baseline & WHtR<0.5 at follow-up | 1.00 | - | 1.00 | - | 1.00 | - |
| WHtR<0.5 at baseline & WHtR≥0.5 at follow-up | 0.98 (0.63, 1.51) | 0.912 | 0.98 (0.64, 1.52) | 0.942 | 0.94 (0.61, 1.46) | 0.797 |
| WHtR≥0.5 at baseline & WHtR<0.5 at follow-up | 1.37 (0.88, 2.12) | 0.163 | 1.37 (0.88, 2.12) | 0.164 | 1.25 (0.81, 1.95) | 0.319 |
| WHtR≥0.5 at baseline & WHtR≥0.5 at follow-up | 1.92 (1.41, 2.61) | <0.001^***^ | 1.93 (1.41, 2.63) | <0.001^***^ | 1.60 (1.17, 2.20) | 0.003^**^ |
| **Dynamic changes of WHR** |  |  |  |  |  |  |
| WHR<0.90 (male)/ 0.85 (female) at baseline & WHR<0.90 (male)/ 0.85 (female) at follow-up | 1.00 | - | 1.00 | - | 1.00 | - |
| WHR<0.90 (male)/ 0.85 (female) at baseline & WHR≥0.90 (male)/ 0.85 (female) at follow-up | 1.34 (0.93, 1.94) | 0.114 | 1.35 (0.94, 1.95) | 0.106 | 1.31 (0.90, 1.88) | 0.155 |
| WHR≥0.90 (male)/ 0.85 (female) at baseline & WHR<0.9 (male)/ 0.85 (female) at follow-up | 1.55 (1.06, 2.26) | 0.023^*^ | 1.57 (1.07, 2.28) | 0.020^*^ | 1.41 (0.97, 2.07) | 0.073 |
| WHR≥0.90 (male)/ 0.85 (female) at baseline & WHR≥0.90 (male)/ 0.85 (female) at follow-up | 2.16 (1.60, 2.90) | <0.001^***^ | 2.16 (1.60, 2.92) | <0.001^***^ | 1.85 (1.37, 2.51) | <0.001^***^ |
| **Dynamic changes of ABSI** |  |  |  |  |  |  |
| ABSI<0.084 at baseline & ABSI<0.084 at follow-up | 1.00 | - | 1.00 | - | 1.00 | - |
| ABSI<0.084 at baseline & ABSI≥0.084 at follow-up | 0.95 (0.77, 1.17) | 0.633 | 0.90 (0.72, 1.11) | 0.310 | 0.91 (0.73, 1.12) | 0.378 |
| ABSI≥0.084 at baseline & ABSI<0.084 at follow-up | 1.15 (0.93, 1.41) | 0.186 | 1.07 (0.86, 1.33) | 0.521 | 1.03 (0.83, 1.28) | 0.778 |
| ABSI≥0.084 at baseline & ABSI≥0.084 at follow-up | 1.03 (0.84, 1.26) | 0.794 | 0.92 (0.73, 1.15) | 0.449 | 0.88 (0.70, 1.10) | 0.267 |
| **Dynamic changes of AVI** |  |  |  |  |  |  |
| AVI<17.5 at baseline & AVI<17.5 at follow-up | 1.00 | - | 1.00 | - | 1.00 | - |
| AVI<17.5 at baseline & AVI≥17.5 at follow-up | 1.44 (1.14, 1.81) | 0.002^**^ | 1.46 (1.16, 1.84) | 0.001^**^ | 1.40 (1.11, 1.77) | 0.004^**^ |
| AVI≥17.5 at baseline & AVI<17.5 at follow-up | 2.10 (1.69, 2.62) | <0.001^***^ | 2.10 (1.69, 2.61) | <0.001^***^ | 1.89 (1.52, 2.37) | <0.001^***^ |
| AVI≥17.5 at baseline & AVI≥17.5 at follow-up | 2.32 (1.97, 2.74) | <0.001^***^ | 2.38 (2.02, 2.80) | <0.001^***^ | 2.15 (1.82, 2.54) | <0.001^***^ |
| **Dynamic changes of BAI** |  |  |  |  |  |  |
| BAI<32.86 at baseline & BAI<32.86 at follow-up | 1.00 | - | 1.00 | - | 1.00 | - |
| BAI<32.86 at baseline & BAI≥32.86 at follow-up | 1.08 (0.84, 1.40) | 0.535 | 1.19 (0.91, 1.56) | 0.194 | 1.12 (0.86, 1.47) | 0.409 |
| BAI≥32.86 at baseline & BAI<32.86 at follow-up | 1.41 (1.11, 1.79) | 0.005^**^ | 1.54 (1.20, 1.99) | <0.001^***^ | 1.35 (1.04, 1.74) | 0.022^*^ |
| BAI≥32.86 at baseline & BAI≥32.86 at follow-up | 1.62 (1.38, 1.91) | <0.001^***^ | 1.83 (1.50, 2.23) | <0.001^***^ | 1.65 (1.35, 2.01) | <0.001^***^ |
| **Dynamic changes of BRI** |  |  |  |  |  |  |
| BRI<5.24 at baseline & BRI<5.24 at follow-up | 1.00 | - | 1.00 | - | 1.00 | - |
| BRI<5.24 at baseline & BRI≥5.24 at follow-up | 1.33 (1.05, 1.68) | 0.017^*^ | 1.37 (1.08, 1.74) | 0.008 | 1.29 (1.02, 1.64) | 0.035^*^ |
| BRI≥5.24 at baseline & BRI<5.24 at follow-up | 1.70 (1.35, 2.14) | <0.001^***^ | 1.76 (1.40, 2.22) | <0.001^***^ | 1.56 (1.23, 1.98) | <0.001^***^ |
| BRI≥5.24 at baseline & BRI≥5.24 at follow-up | 2.10 (1.79, 2.47) | <0.001^***^ | 2.20 (1.85, 2.61) | <0.001^***^ | 1.95 (1.63, 2.32) | <0.001^***^ |
| **Dynamic changes of CI** |  |  |  |  |  |  |
| CI<1.33 at baseline & CI<1.33 at follow-up | 1.00 | - | 1.00 | - | 1.00 | - |
| CI<1.33 at baseline & CI≥1.33 at follow-up | 1.03 (0.83, 1.26) | 0.804 | 1.02 (0.82, 1.25) | 0.888 | 1.00 (0.81, 1.23) | 0.968 |
| CI≥1.33 at baseline & CI<1.33 at follow-up | 1.43 (1.17, 1.75) | <0.001^***^ | 1.41 (1.14, 1.73) | 0.001^**^ | 1.35 (1.09, 1.66) | 0.006^**^ |
| CI≥1.33 at baseline & CI≥1.33 at follow-up | 1.41 (1.17, 1.71) | <0.001^***^ | 1.37 (1.12, 1.69) | 0.003^**^ | 1.28 (1.04, 1.58) | 0.019^*^ |
| **Dynamic changes of WWI** |  |  |  |  |  |  |
| WWI<11.54 at baseline & WWI<11.54 at follow-up | 1.00 | - | 1.00 | - | 1.00 | - |
| WWI<11.54 at baseline & WWI≥11.54 at follow-up | 0.94 (0.74, 1.18) | 0.581 | 0.92 (0.72, 1.17) | 0.486 | 0.89 (0.70, 1.13) | 0.345 |
| WWI≥11.54 at baseline & WWI<11.54 at follow-up | 1.48 (1.20, 1.83) | <0.001^***^ | 1.45 (1.16, 1.82) | 0.001^**^ | 1.34 (1.07, 1.68) | 0.011^*^ |
| WWI≥11.54 at baseline & WWI≥11.54 at follow-up | 1.22 (1.02, 1.47) | 0.029^*^ | 1.18 (0.95, 1.46) | 0.142 | 1.07 (0.86, 1.33) | 0.553 |
| **Dynamic changes of WHHR** |  |  |  |  |  |  |
| WHHR<0.61 at baseline & WHHR<0.61 at follow-up | 1.00 | - | 1.00 | - | 1.00 | - |
| WHHR<0.61 at baseline & WHHR≥0.61 at follow-up | 1.17 (0.94, 1.47) | 0.164 | 1.19 (0.94, 1.50) | 0.142 | 1.14 (0.90, 1.44) | 0.281 |
| WHHR≥0.61 at baseline & WHHR<0.61 at follow-up | 1.26 (1.00, 1.59) | 0.047^*^ | 1.28 (1.01, 1.63) | 0.043^**^ | 1.19 (0.93, 1.51) | 0.165 |
| WHHR≥0.61 at baseline & WHHR≥0.61 at follow-up | 1.49 (1.25, 1.77) | <0.001^***^ | 1.52 (1.24, 1.86) | <0.001^***^ | 1.39 (1.14, 1.71) | 0.002^**^ |

Abbreviations: BMI, body mass index; WC, waist circumference; HC, hip circumference; WHtR, waist-to-height ratio; WHR, waist-to-hip ratio; ABSI, A body shape index; AVI, abdominal volume index; BAI, body adiposity index; BRI, body roundness index; CI, conicity index; WWI, weight-adjusted-waist index; WHHR, waist-hip-height ratio.

Model 1: adjusted by sex, age, smoking status, drinking status.

Model 2: adjusted by model 1 plus TG, TC, HDL, LDL, SBP, DBP.

^*^ *P*-value < 0.05; ^**^ *P* -value < 0.01; ^***^ *P* -value < 0.001.

**Table 9** Cut-off points, area under curve (AUC), sensitivity and specificity of BRI to predict diabetes by sex among hypertensive population.

| **Subgroup** | **Number** | **Cut-off point** | **AUC (95% CI)** | **Sensitivity** | **Specificity** |
| --- | --- | --- | --- | --- | --- |
| Male | 3467 | 3.86 | 0.613 (0.582-0.643) | 0.754 | 0.443 |
| Female | 3523 | 4.92 | 0.638 (0.611-0.666) | 0.643 | 0.586 |
| Pre-menopausal | 764 | 4.01 | 0.589 (0.518-0.660) | 0.700 | 0.470 |
| Post-menopausal | 2759 | 5.08 | 0.639 (0.609-0.669) | 0.648 | 0.587 |
